# Supplementary material for: Extensive cargo identification reveals distinct biological roles of the 12 importin pathways
Source: eLife. 2017 Jan 24;6:e21184. doi: 10.7554/eLife.21184 (PMC5305215; doi:10.7554/eLife.21184)
Supplement: Supplementary file 2. — The direct binding of the candidate cargoes to the NTRs was analyzed by bead halo assay. From well-characterized proteins that have not been reported as cargoes, (i) proteins ranked high (within the top 15% in the 2nd-Z-rankings or 4% in the 3rd-Z-rankings), around presumptive cutoffs (within about top 15–25% in the 2nd-Z-rankings), or lower and (ii) highly ranked proteins that are suspected as indirect cargoes or false positives based on their well-known features, e.g., PMPCA, GALE, UAP1, NQO2, EEF1A2, RAB2A, RAB8A, S100A4, S100A6, S100A13, and S100P, were selected and analyzed. Proteins in (i) verify the cargo identification and cutoff setting, and proteins in (ii) serve for finding indirect cargoes and false positives. The negative rate of these bead halo assays should be higher than the true overall false positive rate of the SILAC-Tp, because proteins in (ii) were selected preferentially. GST or GST-NTR was attached to glutathione-Sepharose beads, mixed with an extract of E. coli expressing GFP or a GFP-fusion protein, and observed by fluorescence microscopy. Q69L-Ran, which inhibits the NTR–cargo functional binding, was added as appropriate. The contrast of the bead fluorescence between the GST and GST-NTR indicates the binding, and the inhibition of this binding by Q69L-Ran certifies the specificity of the binding; ++ or +, positive; ± or –, negative. Summary of the results (p2–5): The results are summarized in both 2nd- and 3rd-Z-rank order. The 2nd-Z-15% and 3rd-Z-4% cargoes are indicated by cyan, and positive binding (++ or +) is indicated by blue. Trn-1 (p6–8): The GFP-fusion proteins were divided into five groups (A–E) according to the expression levels. Because GFP binds weakly to Trn-1, the concentrations of GFP (control) and GFP-fusion proteins were equalized within each group, and the binding was observed in the same conditions. The images are comparable within a group. Trn-2 (p9): GFP weakly binds to Trn-2, and the concentrations of GFP and GFP-fusi [file elife-21184-supp2.pdf]

Supplementary file 2

NTR–cargo direct binding

The direct binding of the candidate cargoes to the NTRs was analyzed by bead halo assay. From well-characterized proteins that have not been reported as cargoes, (i) proteins ranked high (within the top 15% in the 2nd-Z-rankings or 4% in the 3rd-Z-rankings), around presumptive cutoffs (within about top 15–25% in the 2nd-Z-rankings), or lower and (ii) highly ranked proteins that are suspected as indirect cargoes or false positives based on their well-known features, e.g., PMPCA, GALE, UAP1, NQO2, EEF1A2, RAB2A, RAB8A, S100A4, S100A6, S100A13, and S100P, were selected and analyzed. Proteins in (i) verify the cargo identification and cutoff setting, and proteins in (ii) serve for finding indirect cargoes and false positives. The negative rate of these bead halo assays should be higher than the true overall false positive rate of the SILAC-Tp, because proteins in (ii) were selected preferentially. GST or GST-NTR was attached to glutathione-Sepharose beads, mixed with an extract of *E. coli* expressing GFP or a GFP-fusion protein, and observed by fluorescence microscopy. Q69L-Ran, which inhibits the NTR–cargo functional binding, was added as appropriate. The contrast of the bead fluorescence between the GST and GST-NTR indicates the binding, and the inhibition of this binding by Q69L-Ran certifies the specificity of the binding; ++ or +, positive; ± or –, negative.

Summary of the results (p2–5): The results are summarized in both 2nd- and 3rd-Z-rank order. The 2nd-Z-15% and 3rd-Z-4% cargoes are indicated by cyan, and positive binding (++ or +) is indicated by blue.

Trn-1 (p6–8): The GFP-fusion proteins were divided into five groups (A–E) according to the expression levels. Because GFP binds weakly to Trn-1, the concentrations of GFP (control) and GFP-fusion proteins were equalized within each group, and the binding was observed in the same conditions. The images are comparable within a group.

Trn-2 (p9): GFP weakly binds to Trn-2, and the concentrations of GFP and GFP-fusion proteins were equalized. The images are comparable. Proteins whose ranks differed substantially between the Trn-1 and Trn-2 Z-ranking were assayed.

Imp-13 (p10–13): GFP does not bind to Imp-13, and GFP was added to the control mixture at the highest concentration. Three images (GST, GST-Imp-13, and GST-Imp13 + Q69L-Ran) for each GFP-fusion protein were acquired under identical conditions, and the background intensities and dynamic ranges were equalized.

Trn-SR (p14–16): GFP weakly binds to Trn-SR, and the procedures were similar to those used for Trn-1. The GFP-fusion proteins were divided into four groups (A–D), and the images are comparable within a group.

Imp-α/β (p17–20): GFP does not bind to Imp-α or -β, and the procedures were similar to those used for Imp-13. GST-Imp-α_2 lacks the N-terminal Imp-β-binding domain.

Western blotting (p21–22)

The GFP-fusion proteins in the *E. coli* extracts were relatively quantified by Western blotting using an anti-GFP antibody (Roche). The extracts containing the amounts of protein (ng) indicated at the bottoms were loaded. The arrowheads indicate the expected full-length products. The GFP-moieties including those of the partial products were quantified by chemiluminescence. GFP was used as the standard. The Western blots were replicated more than three times.

Accessions and sequences (p23–27)

The cDNAs were cloned from a HeLa cDNA library by PCR. The accession numbers of the proteins are listed. If the sequence of a used protein is different from that in the database, the deleted, substituting, or inserted amino acids are indicated by the colors. The sequences that matched perfectly are not presented.

Summary of Bead Halo Assay (1/4)

2nd-Z-15% or 3rd-Z-4% Cargo | Positive

Trn-1

2nd-Z-Rank Order

Rank by 2nd Z-score (Trn-1)	Rank by 3rd Z-score (Trn-1)	Accession	Gene Name	Direct Binding
1	1	P18846	ATF1	++
3	3	Q9UKD2	MRTO4	++
5	891	Q13509	TUBB3	++
9	5	Q13895	BYSL	++
12		Q96PU8	QKI	++
20		P23511	NFYA	++
26	1156	P06703	S100A6	–
27	1207	Q05639	EEF1A2	–
29	1007	P45973	CBX5	±
33	1182	P49588	AARS	+
47	1086	Q15645	TRIP13	–
51	17	Q15717	ELAVL1	++
63	1190	P62633	CNBP	++
69		O43663	PRC1	–
75	1217	P21291	CSRP1	++
78	1123	O14737	PDCD5	–
108	1173	P26447	S100A4	–
117	838	O60869	EDF1	++
119	24	P56270	MAZ	++
124	47	P41223	BUD31	++
131	1179	Q9UK76	HN1	±
136		Q10713	PMPCA	+
154	153	Q01081	U2AF1	–
161	1037	Q14376	GALE	–
191	1169	Q9Y3F4	STRAP	±
198	749	Q2NL82	TSR1	++
206	31	O00422	SAP18	++
252	684	P22087	FBL	++
266	79	Q6P1J9	CDC73	++
292	283	Q969G3	SMARCE1	++
317	1135	O15446	CD3EAP	++
327	196	Q09161	NCBP1	+
351	111	P62820	RAB1A	±
369		P35232	PHB	±
378	42	Q92922	SMARCC1	–
388	46	P61326	MAGOH	+
629	74	Q13155	AIMP2	++
669	91	P54136	RARS	–
1210	933	Q15651	HMGN3	±
1214	866	Q15233	NONO	++
1242	556	Q9BQ67	GRWD1	±
1325	1066	Q9NV56	MRGBP	–
1390	1029	P61956	SUMO2	–
		Q9UNQ2	DIMT1	++

3rd-Z-Rank Order

Rank by 2nd Z-score (Trn-1)	Rank by 3rd Z-score (Trn-1)	Accession	Gene Name	Direct Binding
1	1	P18846	ATF1	++
3	3	Q9UKD2	MRTO4	++
9	5	Q13895	BYSL	++
51	17	Q15717	ELAVL1	++
119	24	P56270	MAZ	++
206	31	O00422	SAP18	++
378	42	Q92922	SMARCC1	–
388	46	P61326	MAGOH	+
124	47	P41223	BUD31	++
629	74	Q13155	AIMP2	++
266	79	Q6P1J9	CDC73	++
669	91	P54136	RARS	–
351	111	P62820	RAB1A	±
154	153	Q01081	U2AF1	–
327	196	Q09161	NCBP1	+
292	283	Q969G3	SMARCE1	++
1242	556	Q9BQ67	GRWD1	±
252	684	P22087	FBL	++
198	749	Q2NL82	TSR1	++
117	838	O60869	EDF1	++
1214	866	Q15233	NONO	++
5	891	Q13509	TUBB3	++
1210	933	Q15651	HMGN3	±
29	1007	P45973	CBX5	±
1390	1029	P61956	SUMO2	–
161	1037	Q14376	GALE	–
1325	1066	Q9NV56	MRGBP	–
47	1086	Q15645	TRIP13	–
78	1123	O14737	PDCD5	–
317	1135	O15446	CD3EAP	++
26	1156	P06703	S100A6	–
191	1169	Q9Y3F4	STRAP	±
108	1173	P26447	S100A4	–
131	1179	Q9UK76	HN1	±
33	1182	P49588	AARS	+
63	1190	P62633	CNBP	++
27	1207	Q05639	EEF1A2	–
75	1217	P21291	CSRP1	++
12		Q96PU8	QKI	++
20		P23511	NFYA	++
69		O43663	PRC1	–
136		Q10713	PMPCA	+
369		P35232	PHB	±
		Q9UNQ2	DIMT1	++

Trn-2

2nd-Z-Rank Order

Rank by 2nd Z-score (Trn-2)	Rank by 3rd Z-score (Trn-2)	Accession	Gene Name	Direct Binding (Trn-2)	Direct Binding (Trn-1)
11		P35232	PHB	–	±
70		O15446	CD3EAP	+	++
83	769	Q15233	NONO	++	++
542	279	Q6P1J9	CDC73	++	++
594	72	Q9BQ67	GRWD1	–	±
966	297	Q13155	AIMP2	++	++
975	290	P54136	RARS	–	–

3rd-Z-Rank Order

Rank by 2nd Z-score (Trn-2)	Rank by 3rd Z-score (Trn-2)	Accession	Gene Name	Direct Binding (Trn-2)	Direct Binding (Trn-1)
594	72	Q9BQ67	GRWD1	–	±
542	279	Q6P1J9	CDC73	++	++
975	290	P54136	RARS	–	–
966	297	Q13155	AIMP2	++	++
83	769	Q15233	NONO	++	++
11		P35232	PHB	–	±
70		O15446	CD3EAP	+	++

Summary of Bead Halo Assay (2/4)

2nd-Z-15% or 3rd-Z-4% Cargo — Positive

Imp-13

2nd-Z-Rank Order

Rank by 2nd Z-score (Imp-13)	Rank by 3rd Z-score (Imp-13)	Accession	Gene Name	Direct Binding
3	939	P25815	S100P	–
4	409	Q14558	PRPSAP1	++
5	2	Q96AG4	LRRC59	++
6	3	P52657	GTF2A2	++
8	4	P61326	MAGOH	++
10	10	Q9Y3T9	NOC2L	++
11		Q9BTZ2	DHRS4	++
12	8	Q96GQ7	DDX27	+
13	285	O15446	CD3EAP	–
14	777	Q16222	UAP1	–
15	1307	Q14376	GALE	–
16	1198	P07951	TPM2	–
17		Q8NEM2	SHCBP1	–
18	37	Q8WUB8	PHF10	–
19	6	Q9NRG0	CHRAC1	++
23	7	Q9NRF9	POLE3	–
29		P08559	PDHA1	++
32		P37268	FDFT1	+
33	9	P23511	NFYA	++
42	1352	Q7L0Y3	TRMT10C	++
46		Q92747	ARPC1A	++
50	1657	P46937	YAP1	–
54	215	O14737	PDCD5	++
55	1130	O60869	EDF1	±
59		O75368	SH3BGRL	–
60	11	P16083	NQO2	–
61	717	P26447	S100A4	–
67	307	P49588	AARS	–
72	138	Q96IZ0	PAWR	++
77	534	Q9UK76	HN1	–
79		Q9GZR2	REXO4	–
82	1328	P62633	CNBP	++
87	163	P06703	S100A6	–
88	547	P21291	CSRP1	++
94	100	Q15717	ELAVL1	++
96	466	Q9Y3F4	STRAP	+
118	24	P61956	SUMO2	–
132	33	O43663	PRC1	++
136	1153	Q13573	SNW1	++
189	48	Q99584	S100A13	–
195	38	Q9H0A0	NAT10	–
233	52	Q9BQ67	GRWD1	+
234	29	Q969G3	SMARCE1	++
253	978	P61106	RAB14	+
263	1335	Q9BTL3	FAM103A1	++
326	153	P41223	BUD31	++
392	902	P18858	LIG1	++
417	193	Q15645	TRIP13	–
470	102	P18754	RCC1	–
474		Q99661	KIF2C	±
535	155	Q10713	PMPCA	–
587	157	Q14137	BOP1	–
594	182	Q09161	NCBP1	±
595	198	Q86U70	LDB1	++
598	126	O43148	RNMT	–
602	87	P52298	NCBP2	++
639	1539	Q9NQP4	PFDN4	–
835	376	Q99567	NUP88	±
858	348	P22087	FBL	–
859	252	Q9UKD2	MRTO4	–
866	202	P78347	GTF2I	+
1295	1628	Q13330	MTA1	++
1535	835	P54727	RAD23B	–
1657	1150	Q8N7H5	PAF1	±

3rd-Z-Rank Order

Rank by 2nd Z-score (Imp-13)	Rank by 3rd Z-score (Imp-13)	Accession	Gene Name	Direct Binding
5	2	Q96AG4	LRRC59	++
6	3	P52657	GTF2A2	++
8	4	P61326	MAGOH	++
19	6	Q9NRG0	CHRAC1	++
23	7	Q9NRF9	POLE3	–
12	8	Q96GQ7	DDX27	+
33	9	P23511	NFYA	++
10	10	Q9Y3T9	NOC2L	++
60	11	P16083	NQO2	–
118	24	P61956	SUMO2	–
234	29	Q969G3	SMARCE1	++
132	33	O43663	PRC1	++
18	37	Q8WUB8	PHF10	–
195	38	Q9H0A0	NAT10	–
189	48	Q99584	S100A13	–
233	52	Q9BQ67	GRWD1	+
602	87	P52298	NCBP2	++
94	100	Q15717	ELAVL1	++
470	102	P18754	RCC1	–
598	126	O43148	RNMT	–
72	138	Q96IZ0	PAWR	++
326	153	P41223	BUD31	++
535	155	Q10713	PMPCA	–
587	157	Q14137	BOP1	–
87	163	P06703	S100A6	–
594	182	Q09161	NCBP1	±
417	193	Q15645	TRIP13	–
595	198	Q86U70	LDB1	++
866	202	P78347	GTF2I	+
54	215	O14737	PDCD5	++
859	252	Q9UKD2	MRTO4	–
13	285	O15446	CD3EAP	–
67	307	P49588	AARS	–
858	348	P22087	FBL	–
835	376	Q99567	NUP88	±
4	409	Q14558	PRPSAP1	++
96	466	Q9Y3F4	STRAP	+
77	534	Q9UK76	HN1	–
88	547	P21291	CSRP1	++
61	717	P26447	S100A4	–
14	777	Q16222	UAP1	–
1535	835	P54727	RAD23B	–
392	902	P18858	LIG1	++
3	939	P25815	S100P	–
253	978	P61106	RAB14	+
55	1130	O60869	EDF1	±
1657	1150	Q8N7H5	PAF1	±
136	1153	Q13573	SNW1	++
16	1198	P07951	TPM2	–
15	1307	Q14376	GALE	–
82	1328	P62633	CNBP	++
263	1335	Q9BTL3	FAM103A1	++
42	1352	Q7L0Y3	TRMT10C	++
639	1539	Q9NQP4	PFDN4	–
1295	1628	Q13330	MTA1	++
50	1657	P46937	YAP1	–
11		Q9BTZ2	DHRS4	++
17		Q8NEM2	SHCBP1	–
29		P08559	PDHA1	++
32		P37268	FDFT1	+
46		Q92747	ARPC1A	++
59		O75368	SH3BGRL	–
79		Q9GZR2	REXO4	–
474		Q99661	KIF2C	±

Summary of Bead Halo Assay (3/4)

2nd-Z-15% or 3rd-Z-4% Cargo | Positive

Trn-SR

2nd-Z-Rank Order

Rank by 2nd Z-score (Trn-SR)	Rank by 3rd Z-score (Trn-SR)	Accession	Gene Name	Direct Binding
1	1386	Q10713	PMPCA	±
10	3	Q8IWS0	PHF6	++
12		Q99661	KIF2C	–
14	21	Q8N7H5	PAF1	–
36		Q96GQ7	DDX27	++
37	29	P83916	CBX1	–
74	36	Q9H0A0	NAT10	–
87	23	O00422	SAP18	++
98	20	P53999	SUB1	–
106	94	P22087	FBL	++
112	469	Q14137	BOP1	–
116	58	P17844	DDX5	++
123	25	P18615	NELFE	++
155	1144	Q16222	UAP1	–
176	241	Q08945	SSRP1	±
190	1557	P04181	OAT	+
198	33	P25685	DNAJB1	+
202	63	Q01081	U2AF1	–
206	467	Q6P1J9	CDC73	++
209	312	Q13573	SNW1	++
211		P61019	RAB2A	–
222	814	Q96PU8	QKI	–
237	65	Q86U70	LDB1	+
277	1591	P25815	S100P	–
279	494	Q9UKD2	MRTO4	–
320	62	P61006	RAB8A	±
390	117	P56270	MAZ	++
398	87	Q9NQP4	PFDN4	–
416	53	Q96ES7	CCDC101	++
491	74	P08559	PDHA1	+
510	81	O43663	PRC1	±
521	120	P18754	RCC1	–
644	147	P46063	RECQL	–
702	198	P41223	BUD31	++
783	130	Q13155	AIMP2	+

3rd-Z-Rank Order

Rank by 2nd Z-score (Trn-SR)	Rank by 3rd Z-score (Trn-SR)	Accession	Gene Name	Direct Binding
10	3	Q8IWS0	PHF6	++
98	20	P53999	SUB1	–
14	21	Q8N7H5	PAF1	–
87	23	O00422	SAP18	++
123	25	P18615	NELFE	++
37	29	P83916	CBX1	–
198	33	P25685	DNAJB1	+
74	36	Q9H0A0	NAT10	–
416	53	Q96ES7	CCDC101	++
116	58	P17844	DDX5	++
320	62	P61006	RAB8A	±
202	63	Q01081	U2AF1	–
237	65	Q86U70	LDB1	+
491	74	P08559	PDHA1	+
510	81	O43663	PRC1	±
398	87	Q9NQP4	PFDN4	–
106	94	P22087	FBL	++
390	117	P56270	MAZ	++
521	120	P18754	RCC1	–
783	130	Q13155	AIMP2	+
644	147	P46063	RECQL	–
702	198	P41223	BUD31	++
176	241	Q08945	SSRP1	±
209	312	Q13573	SNW1	++
206	467	Q6P1J9	CDC73	++
112	469	Q14137	BOP1	–
279	494	Q9UKD2	MRTO4	–
222	814	Q96PU8	QKI	–
155	1144	Q16222	UAP1	–
1	1386	Q10713	PMPCA	±
190	1557	P04181	OAT	+
277	1591	P25815	S100P	–
12		Q99661	KIF2C	–
36		Q96GQ7	DDX27	++
211		P61019	RAB2A	–

Summary of Bead Halo Assay (4/4)

2nd-Z-15% or 3rd-Z-4% Cargo | Positive

Imp-α/β

2nd-Z-Rank Order

Rank by 2nd Z-score (Imp-β)	Rank by 3rd Z-score (Imp-β)	Accession	Gene Name	Imp-α Direct Binding	Imp-β Direct Binding
2	2	O43148	RNMT	++	–
3	3	Q9BTL3	FAM103A1	–	++
5	5	Q2NL82	TSR1	+	++
10		P07305	H1F0	++	–
16	1503	Q9BV86	NTMT1	–	–
19	7	P52298	NCBP2	+	–
20	6	Q96AG4	LRRC59	–	–
25	8	Q09161	NCBP1	++	–
28	1486	Q7L0Y3	TRMT10C	++	–
30	12	Q99567	NUP88	–	–
31		P23511	NFYA	++	++
33	21	Q13330	MTA1	++	++
39	1342	Q14558	PRPSAP1	++	–
46	18	P18754	RCC1	++	–
52	1048	P16083	NQO2	–	–
62	892	Q13573	SNW1	++	–
63	1191	Q16222	UAP1	–	–
66	31	Q86U70	LDB1	++	–
68	86	P17844	DDX5	++	++
74		Q99661	KIF2C	++	–
75	63	P04181	OAT	–	–
78		Q96GQ7	DDX27	++	–
79	61	Q9NV56	MRGBP	++	–
80	40	P49005	POLD2	–	–
94	25	P49711	CTCF	++	++
96		O15446	CD3EAP	++	–
97	39	Q969G3	SMARCE1	++	++
109	30	P56270	MAZ	++	++
135	1603	Q10713	PMPCA	–	–
151	1203	O43663	PRC1	++	–
152	1299	P25815	S100P	–	–
155	42	Q9Y657	SPIN1	++	++
156	1579	P35232	PHB	–	–
158	78	Q15717	ELAVL1	+	–
180	44	P17096	HMGA1	++	–
188	52	Q15651	HMGN3	++	–
190	1134	Q01081	U2AF1	–	–
193	524	P53999	SUB1	++	–
238	351	P42166	TMPO	++	–
248	992	Q8IWS0	PHF6	++	–
259	234	Q8IZ73	RPUSD2	++	++
276		Q9GZR2	REXO4	++	–
304		Q8NEM2	SHCBP1	–	–
382	96	P22087	FBL	++	++
436	196	P25685	DNAJB1	++	–
450	155	O00422	SAP18	++	–
470	403	P46063	RECQL	++	–
529	585	Q9NRF9	POLE3	–	–
573	237	Q9Y3T9	NOC2L	++	–
674	1476	Q14137	BOP1	++	–
693	1466	P07951	TPM2	–	–
698	675	P18858	LIG1	++	–
711	204	Q15233	NONO	++	±
768	210	P61956	SUMO2	–	–
781	261	P61006	RAB8A	–	–
782	424	Q9NRG0	CHRAC1	–	–
930	1597	Q9H0A0	NAT10	++	–
1332	994	Q99575	POP1	++	–
1490	954	Q99584	S100A13	–	–
1494	819	Q96IZ0	PAWR	–	–
		Q9BTZ2	DHRS4	–	++

3rd-Z-Rank Order

Rank by 2nd Z-score (Imp-β)	Rank by 3rd Z-score (Imp-β)	Accession	Gene Name	Imp-α Direct Binding	Imp-β Direct Binding
2	2	O43148	RNMT	++	–
3	3	Q9BTL3	FAM103A1	–	++
5	5	Q2NL82	TSR1	+	++
20	6	Q96AG4	LRRC59	–	–
19	7	P52298	NCBP2	+	–
25	8	Q09161	NCBP1	++	–
30	12	Q99567	NUP88	–	–
46	18	P18754	RCC1	++	–
33	21	Q13330	MTA1	++	++
94	25	P49711	CTCF	++	++
109	30	P56270	MAZ	++	++
66	31	Q86U70	LDB1	++	–
97	39	Q969G3	SMARCE1	++	++
80	40	P49005	POLD2	–	–
155	42	Q9Y657	SPIN1	++	++
180	44	P17096	HMGA1	++	–
188	52	Q15651	HMGN3	++	–
79	61	Q9NV56	MRGBP	++	–
75	63	P04181	OAT	–	–
158	78	Q15717	ELAVL1	+	–
68	86	P17844	DDX5	++	++
382	96	P22087	FBL	++	++
450	155	O00422	SAP18	++	–
436	196	P25685	DNAJB1	++	–
711	204	Q15233	NONO	++	±
768	210	P61956	SUMO2	–	–
259	234	Q8IZ73	RPUSD2	++	++
573	237	Q9Y3T9	NOC2L	++	–
781	261	P61006	RAB8A	–	–
238	351	P42166	TMPO	++	–
470	403	P46063	RECQL	++	–
782	424	Q9NRG0	CHRAC1	–	–
193	524	P53999	SUB1	++	–
529	585	Q9NRF9	POLE3	–	–
698	675	P18858	LIG1	++	–
1494	819	Q96IZ0	PAWR	–	–
62	892	Q13573	SNW1	++	–
1490	954	Q99584	S100A13	–	–
248	992	Q8IWS0	PHF6	++	–
1332	994	Q99575	POP1	++	–
52	1048	P16083	NQO2	–	–
190	1134	Q01081	U2AF1	–	–
63	1191	Q16222	UAP1	–	–
151	1203	O43663	PRC1	++	–
152	1299	P25815	S100P	–	–
39	1342	Q14558	PRPSAP1	++	–
693	1466	P07951	TPM2	–	–
674	1476	Q14137	BOP1	++	–
28	1486	Q7L0Y3	TRMT10C	++	–
16	1503	Q9BV86	NTMT1	–	–
156	1579	P35232	PHB	–	–
930	1597	Q9H0A0	NAT10	++	–
135	1603	Q10713	PMPCA	–	–
10		P07305	H1F0	++	–
31		P23511	NFYA	++	++
74		Q99661	KIF2C	++	–
78		Q96GQ7	DDX27	++	–
96		O15446	CD3EAP	++	–
276		Q9GZR2	REXO4	++	–
304		Q8NEM2	SHCBP1	–	–
		Q9BTZ2	DHRS4	–	++

Trn-1 (1/3)

A

Rank by 2nd Z-score	Rank by 3rd Z-score	Accession	Gene Name	Direct Binding
			GFP	–
3	3	Q9UKD2	MRTO4	++
9	5	Q13895	BYSL	++
12		Q96PU8	QKI	++
		Q9UNQ2	DIMT1	++

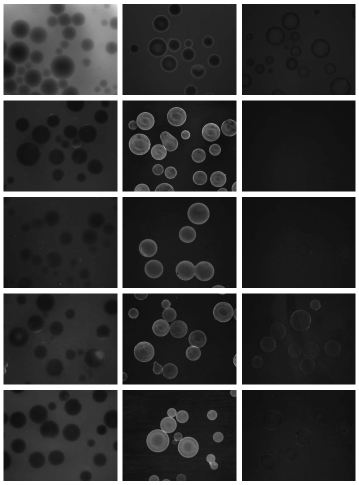

B

Rank by 2nd Z-score	Rank by 3rd Z-score	Accession	Gene Name	Direct Binding
			GFP	–
26	1156	P06703	S100A6	–
63	1190	P62633	CNBP	++
75	1217	P21291	CSRP1	++
78	1123	O14737	PDCD5	–
108	1173	P26447	S100A4	–
117	838	O60869	EDF1	++
124	47	P41223	BUD31	++
1390	1029	P61956	SUMO2	–

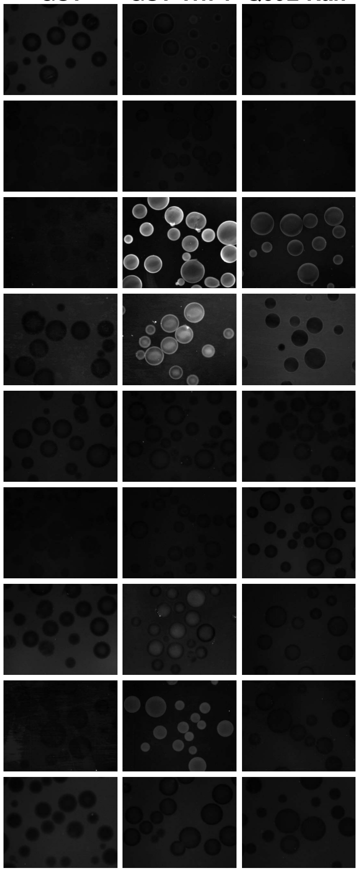

Trn-1 (2/3)

C

Rank by 2nd Z-score	Rank by 3rd Z-score	Accession	Gene Name	Direct Binding
			GFP	–
1	1	P18846	ATF1	++
20		P23511	NFYA	++
29	1007	P45973	CBX5	±
47	1086	Q15645	TRIP13	–
51	17	Q15717	ELAVL1	++
119	24	P56270	MAZ	++
131	1179	Q9UK76	HN1	±
198	749	Q2NL82	TSR1	++
206	31	O00422	SAP18	++
252	684	P22087	FBL	++
292	283	Q969G3	SMARCE1	++
317	1135	O15446	CD3EAP	++
378	42	Q92922	SMARCC1	–
629	74	Q13155	AIMP2	++
1210	933	Q15651	HMGN3	±
1242	556	Q9BQ67	GRWD1	±
1325	1066	Q9NV56	MRGBP	–

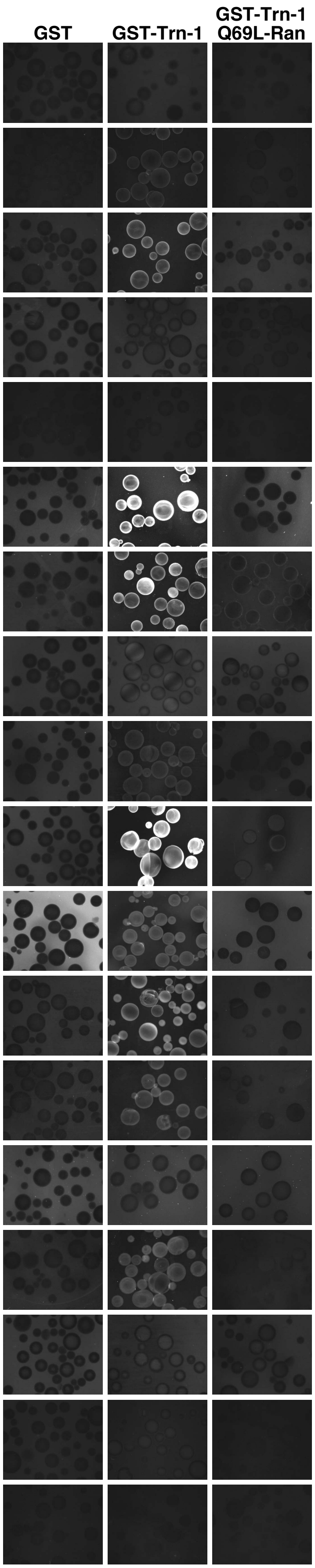

Bead Halo Assay (3/15)

Trn-1 (3/3)

D

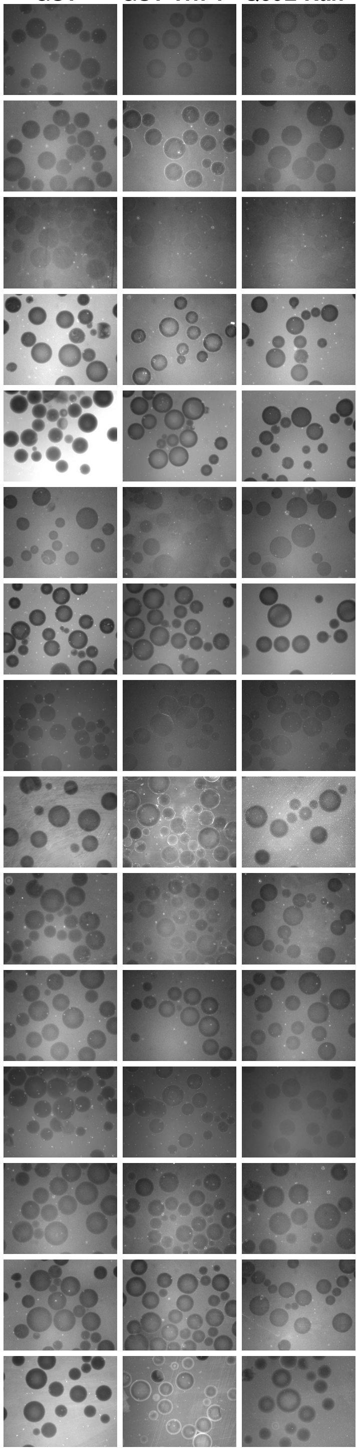

Rank by 2nd Z-score	Rank by 3rd Z-score	Accession	Gene Name	Direct Binding
			GFP	–
5	891	Q13509	TUBB3	++
27	1207	Q05639	EEF1A2	–
33	1182	P49588	AARS	+
69		O43663	PRC1	–
154	153	Q01081	U2AF1	–
161	1037	Q14376	GALE	–
191	1169	Q9Y3F4	STRAP	±
266	79	Q6P1J9	CDC73	++
327	196	Q09161	NCBP1	+
351	111	P62820	RAB1A	±
369		P35232	PHB	±
388	46	P61326	MAGOH	+
669	91	P54136	RARS	–
1214	866	Q15233	NONO	++

E

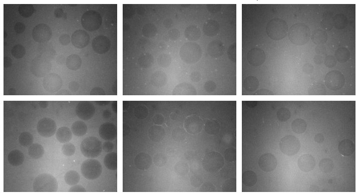

Rank by 2nd Z-score	Rank by 3rd Z-score	Accession	Gene Name	Direct Binding
			GFP	–
136		Q10713	PMPCA	+

Bead Halo Assay (4/15)

Trn-2 (1/1)

Rank by 2nd Z-score	Rank by 3rd Z-score	Accession	Gene Name	GST	GST-Trn-2	GST-Trn-2 Q69L-Ran	Direct Binding
			GFP	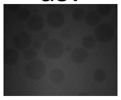	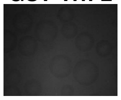	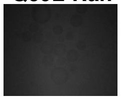	–
11		P35232	PHB	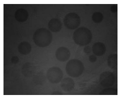	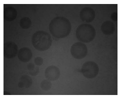	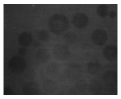	–
70		O15446	CD3EAP	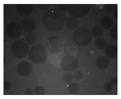	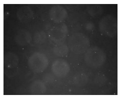	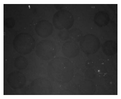	+
83	769	Q15233	NONO	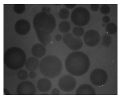	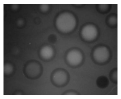	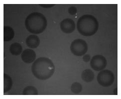	++
542	279	Q6P1J9	CDC73	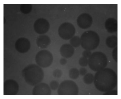	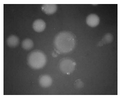	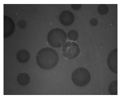	++
594	72	Q9BQ67	GRWD1	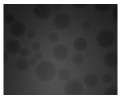	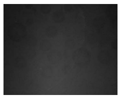	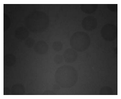	–
966	297	Q13155	AIMP2	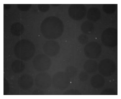	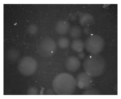	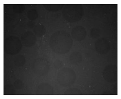	++
975	290	P54136	RARS	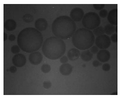	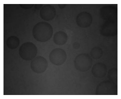	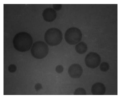	–

Bead Halo Assay (5/15)

Imp-13 (1/4)

Rank by 2nd Z-score	Rank by 3rd Z-score	Accession	Gene Name	GST	GST-Imp-13	GST-Imp-13 Q69L-Ran	Direct Binding
			GFP				–
3	939	P25815	S100P				–
4	409	Q14558	PRPSAP1				++
5	2	Q96AG4	LRRC59				++
6	3	P52657	GTF2A2				++
8	4	P61326	MAGOH				++
10	10	Q9Y3T9	NOC2L				++
11		Q9BTZ2	DHRS4				++
12	8	Q96GQ7	DDX27				+
13	285	O15446	CD3EAP				–
14	777	Q16222	UAP1				–
15	1307	Q14376	GALE				–
16	1198	P07951	TPM2				–
17		Q8NEM2	SHCBP1				–
18	37	Q8WUB8	PHF10				–
19	6	Q9NRG0	CHRAC1				++
23	7	Q9NRF9	POLE3				–
29		P08559	PDHA1				++
32		P37268	FDFT1				+
33	9	P23511	NFYA				++

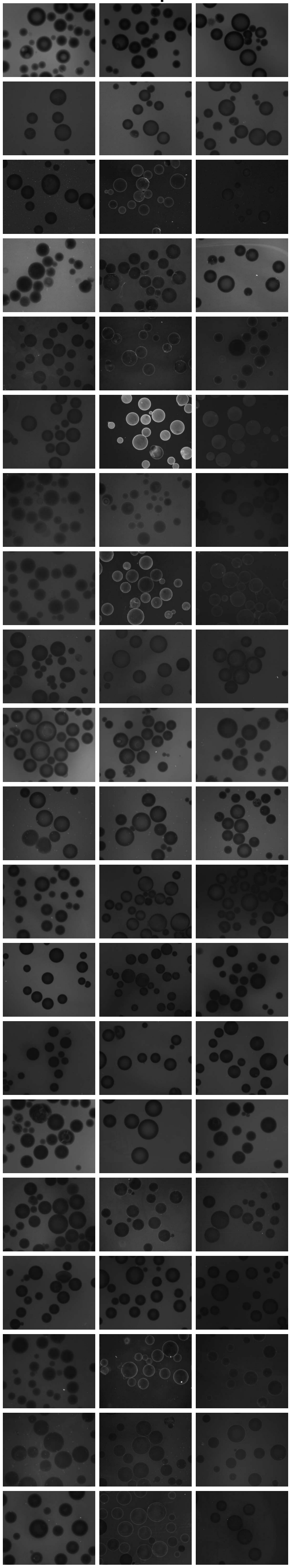

Bead Halo Assay (6/15)

Imp-13 (2/4)

Rank by 2nd Z-score	Rank by 3rd Z-score	Accession	Gene Name	GST	GST-Imp-13	GST-Imp-13 Q69L-Ran	Direct Binding
42	1352	Q7L0Y3	TRMT10C	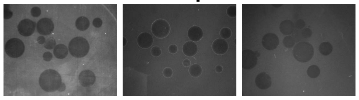			++
46		Q92747	ARPC1A	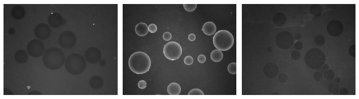			++
50	1657	P46937	YAP1	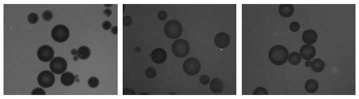			–
54	215	O14737	PDCD5	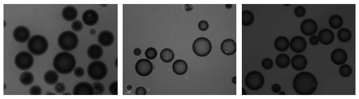			++
55	1130	O60869	EDF1	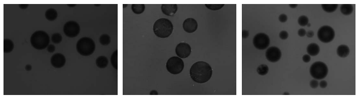			±
59		O75368	SH3BGRL	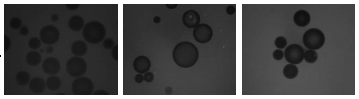			–
60	11	P16083	NQO2	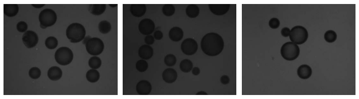			–
61	717	P26447	S100A4	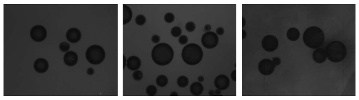			–
67	307	P49588	AARS	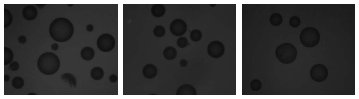			–
72	138	Q96IZ0	PAWR	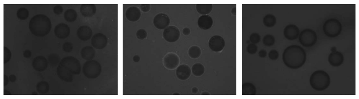			++
77	534	Q9UK76	HN1	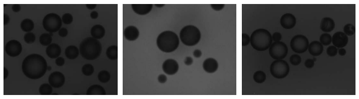			–
79		Q9GZR2	REXO4	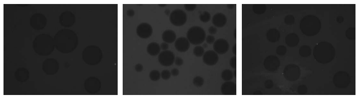			–
82	1328	P62633	CNBP	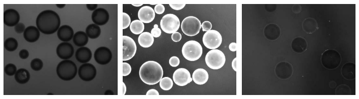			++
87	163	P06703	S100A6	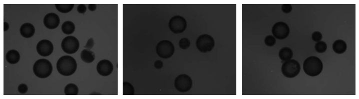			–
88	547	P21291	CSRP1	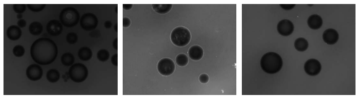			++
94	100	Q15717	ELAVL1	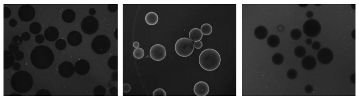			++
96	466	Q9Y3F4	STRAP	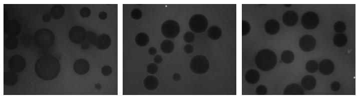			+
118	24	P61956	SUMO2	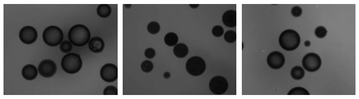			–
132	33	O43663	PRC1	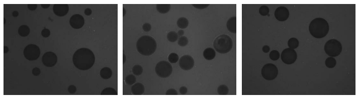			++
136	1153	Q13573	SNW1	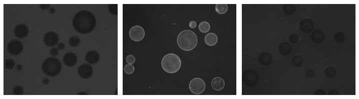			++

Bead Halo Assay (7/15)

Imp-13 (3/4)

Rank by 2nd Z-score	Rank by 3rd Z-score	Accession	Gene Name	GST	GST-Imp-13	GST-Imp-13 Q69L-Ran	Direct Binding
189	48	Q99584	S100A13	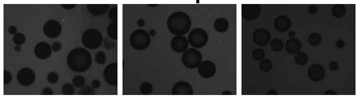			–
195	38	Q9H0A0	NAT10	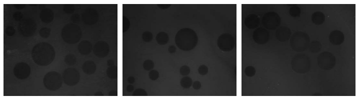			–
233	52	Q9BQ67	GRWD1	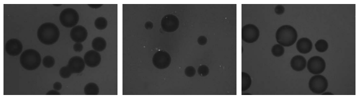			+
234	29	Q969G3	SMARCE1	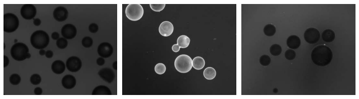			++
253	978	P61106	RAB14	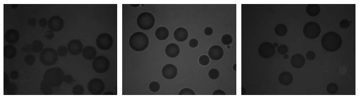			+
263	1335	Q9BTL3	FAM103A1	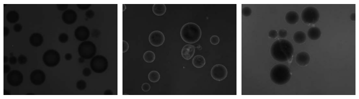			++
326	153	P41223	BUD31	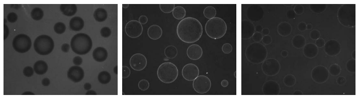			++
392	902	P18858	LIG1	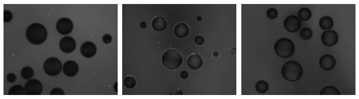			++
417	193	Q15645	TRIP13	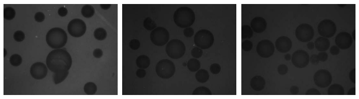			–
470	102	P18754	RCC1	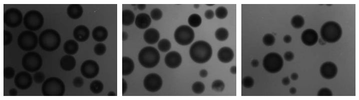			–
474		Q99661	KIF2C	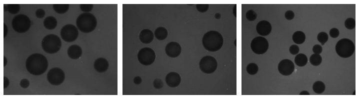			±
535	155	Q10713	PMPCA	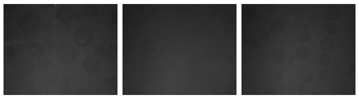			–
587	157	Q14137	BOP1	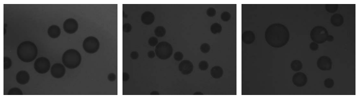			–
594	182	Q09161	NCBP1	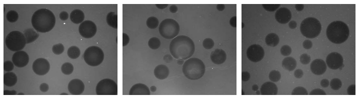			±
595	198	Q86U70	LDB1	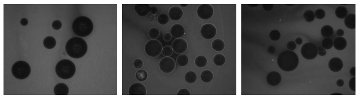			++
598	126	O43148	RNMT	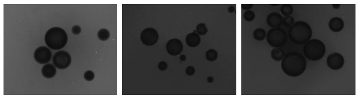			–
602	87	P52298	NCBP2	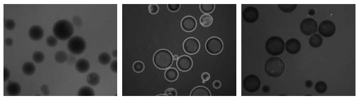			++
639	1539	Q9NQP4	PFDN4	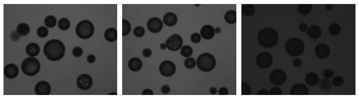			–
835	376	Q99567	NUP88	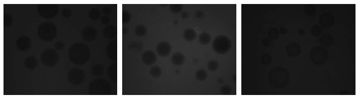			±
858	348	P22087	FBL	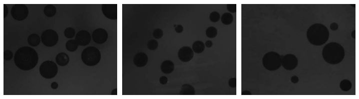			–

Bead Halo Assay (8/15)

Imp-13 (4/4)

Rank by 2nd Z-score	Rank by 3rd Z-score	Accession	Gene Name	GST	GST-Imp-13	GST-Imp-13 Q69L-Ran	Direct Binding
859	252	Q9UKD2	MRTO4	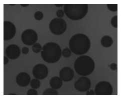	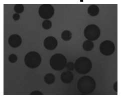	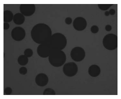	–
866	202	P78347	GTF2I	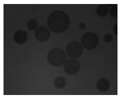	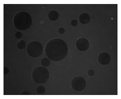	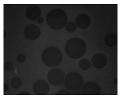	+
1295	1628	Q13330	MTA1	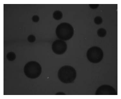	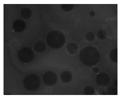	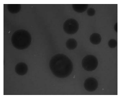	++
1535	835	P54727	RAD23B	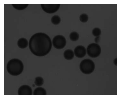	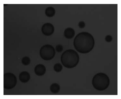	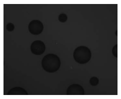	–
1657	1150	Q8N7H5	PAF1	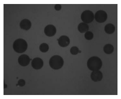	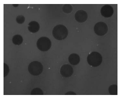	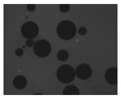	±

Trn-SR (1/3)

A

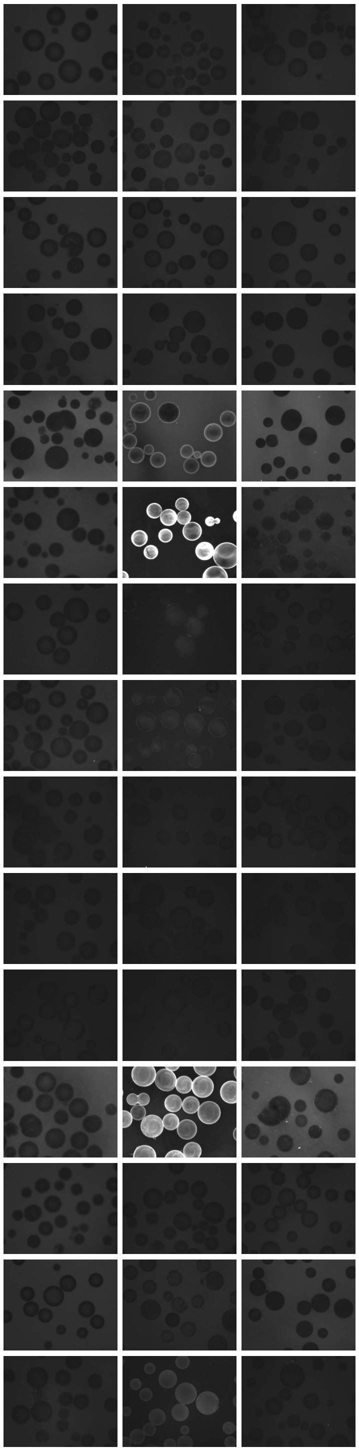

Rank by 2nd Z-score	Rank by 3rd Z-score	Accession	Gene Name	GST	GST-Trn-SR	GST-Trn-SR Q69L-Ran	Direct Binding
			GFP				–
14	21	Q8N7H5	PAF1				–
37	29	P83916	CBX1				–
98	20	P53999	SUB1				–
106	94	P22087	FBL				++
123	25	P18615	NELFE				++
190	1557	P04181	OAT				+
198	33	P25685	DNAJB1				+
222	814	Q96PU8	QKI				–
277	1591	P25815	S100P				–
279	494	Q9UKD2	MRTO4				–
390	117	P56270	MAZ				++
398	87	Q9NQP4	PFDN4				–
521	120	P18754	RCC1				–
702	198	P41223	BUD31				++

Trn-SR (2/3)

B

Rank by 2nd Z-score	Rank by 3rd Z-score	Accession	Gene Name	Direct Binding
			GFP	–
10	3	Q8IWS0	PHF6	++
36		Q96GQ7	DDX27	++
87	23	O00422	SAP18	++
112	469	Q14137	BOP1	–
155	1144	Q16222	UAP1	–
176	241	Q08945	SSRP1	±
209	312	Q13573	SNW1	++
211		P61019	RAB2A	–
237	65	Q86U70	LDB1	+
416	53	Q96ES7	CCDC101	++
510	81	O43663	PRC1	±
644	147	P46063	RECQL	–
783	130	Q13155	AIMP2	+

GST | GST-Trn-SR | GST-Trn-SR Q69L-Ran

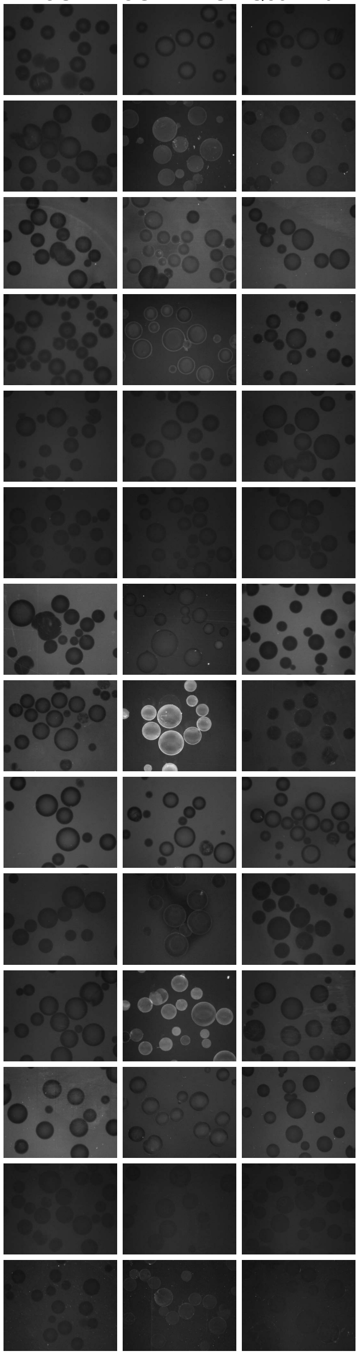

Trn-SR (3/3)

C

Rank by 2nd Z-score	Rank by 3rd Z-score	Accession	Gene Name	GST	GST-Trn-SR	GST-Trn-SR Q69L-Ran	Direct Binding
			GFP				–
12		Q99661	KIF2C				–
74	36	Q9H0A0	NAT10				–
116	58	P17844	DDX5				++
202	63	Q01081	U2AF1				–
206	467	Q6P1J9	CDC73				++
320	62	P61006	RAB8A				±
491	74	P08559	PDHA1				+

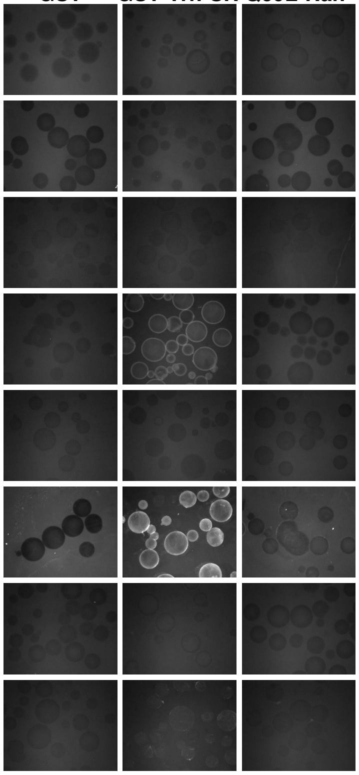

D

Rank by 2nd Z-score	Rank by 3rd Z-score	Accession	Gene Name	GST	GST-Trn-SR	GST-Trn-SR Q69L-Ran	Direct Binding
			GFP				–
1	1386	Q10713	PMPCA				±

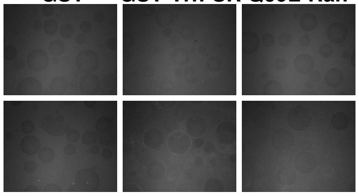

Bead Halo Assay (12/15)

Imp-α/β (1/4)

GST | GST-Imp-α2ΔN | GST-Imp-β | GST-Imp-β Q69L-Ran

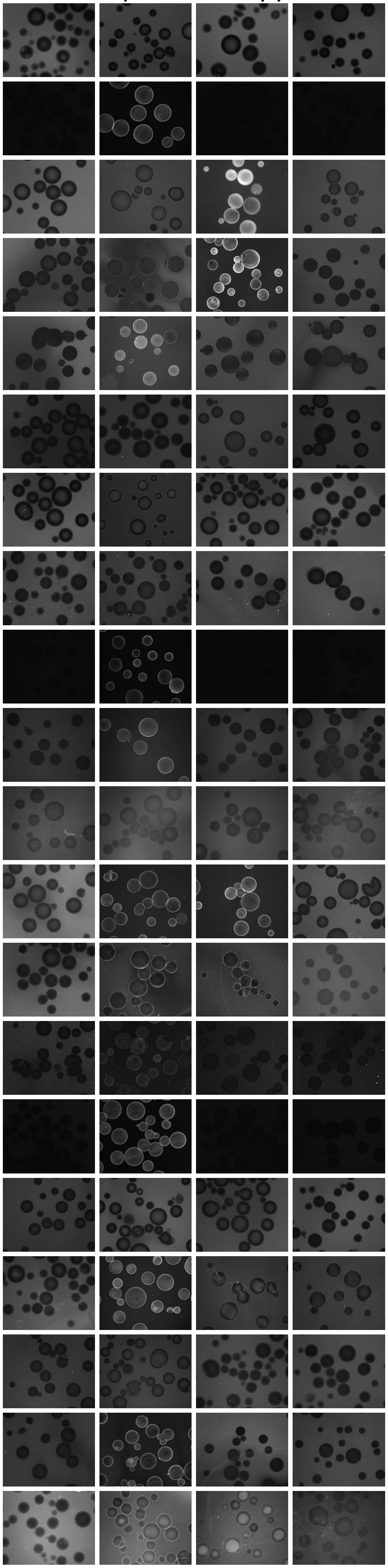

Rank by 2nd Z-score	Rank by 3rd Z-score	Accession	Gene Name	Imp-α Direct Binding	Imp-β Direct Binding
			GFP	–	–
2	2	O43148	RNMT	++	–
3	3	Q9BTL3	FAM103A1	–	++
5	5	Q2NL82	TSR1	+	++
10		P07305	H1F0	++	–
16	1503	Q9BV86	NTMT1	–	–
19	7	P52298	NCBP2	+	–
20	6	Q96AG4	LRRC59	–	–
25	8	Q09161	NCBP1	++	–
28	1486	Q7L0Y3	TRMT10C	++	–
30	12	Q99567	NUP88	–	–
31		P23511	NFYA	++	++
33	21	Q13330	MTA1	++	++
39	1342	Q14558	PRPSAP1	++	–
46	18	P18754	RCC1	++	–
52	1048	P16083	NQO2	–	–
62	892	Q13573	SNW1	++	–
63	1191	Q16222	UAP1	–	–
66	31	Q86U70	LDB1	++	–
68	86	P17844	DDX5	++	++

Imp-α/β (2/4)

Rank by 2nd Z-score	Rank by 3rd Z-score	Accession	Gene Name	GST	GST-Imp-α2ΔN	GST-Imp-β	GST-Imp-β Q69L-Ran	Imp-α Direct Binding	Imp-β Direct Binding
74		Q99661	KIF2C					++	–
75	63	P04181	OAT					–	–
78		Q96GQ7	DDX27					++	–
79	61	Q9NV56	MRGBP					++	–
80	40	P49005	POLD2					–	–
94	25	P49711	CTCF					++	++
96		O15446	CD3EAP					++	–
97	39	Q969G3	SMARCE1					++	++
109	30	P56270	MAZ					++	++
135	1603	Q10713	PMPCA					–	–
151	1203	O43663	PRC1					++	–
152	1299	P25815	S100P					–	–
155	42	Q9Y657	SPIN1					++	++
156	1579	P35232	PHB					–	–
158	78	Q15717	ELAVL1					+	–
180	44	P17096	HMGA1					++	–
188	52	Q15651	HMGN3					++	–
190	1134	Q01081	U2AF1					–	–
193	524	P53999	SUB1					++	–
238	351	P42166	TMPO					++	–

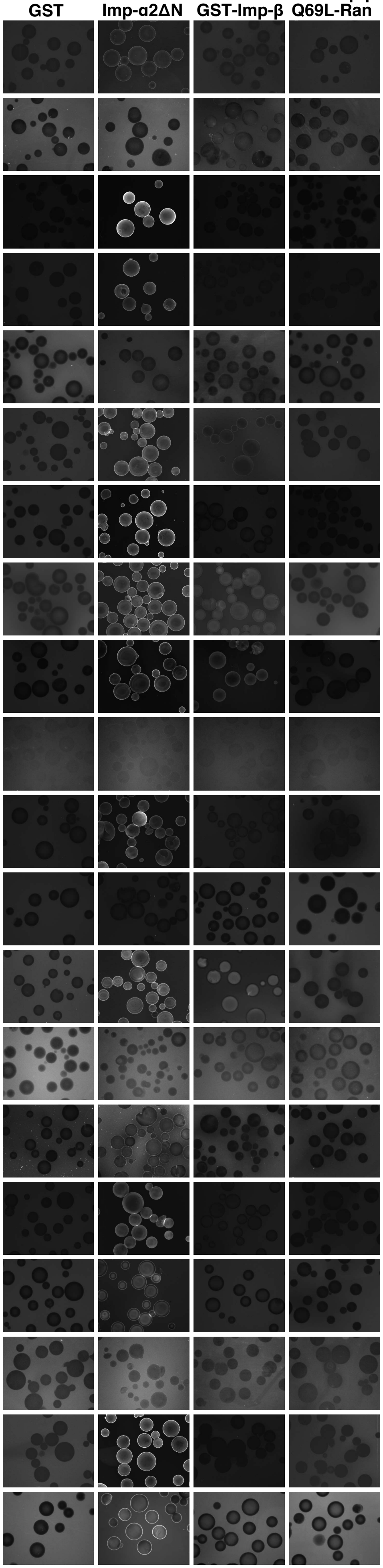

Bead Halo Assay (14/15)

Imp-α/β (3/4)

Rank by 2nd Z-score	Rank by 3rd Z-score	Accession	Gene Name	Imp-α Direct Binding	Imp-β Direct Binding
248	992	Q8IWS0	PHF6	++	–
259	234	Q8IZ73	RPUSD2	++	++
276		Q9GZR2	REXO4	++	–
304		Q8NEM2	SHCBP1	–	–
382	96	P22087	FBL	++	++
436	196	P25685	DNAJB1	++	–
450	155	O00422	SAP18	++	–
470	403	P46063	RECQL	++	–
529	585	Q9NRF9	POLE3	–	–
573	237	Q9Y3T9	NOC2L	++	–
674	1476	Q14137	BOP1	++	–
693	1466	P07951	TPM2	–	–
698	675	P18858	LIG1	++	–
711	204	Q15233	NONO	++	±
768	210	P61956	SUMO2	–	–
781	261	P61006	RAB8A	–	–
782	424	Q9NRG0	CHRAC1	–	–
930	1597	Q9H0A0	NAT10	++	–
1332	994	Q99575	POP1	++	–
1490	954	Q99584	S100A13	–	–

GST | GST-Imp-α2ΔN | GST-Imp-β | GST-Imp-β Q69L-Ran

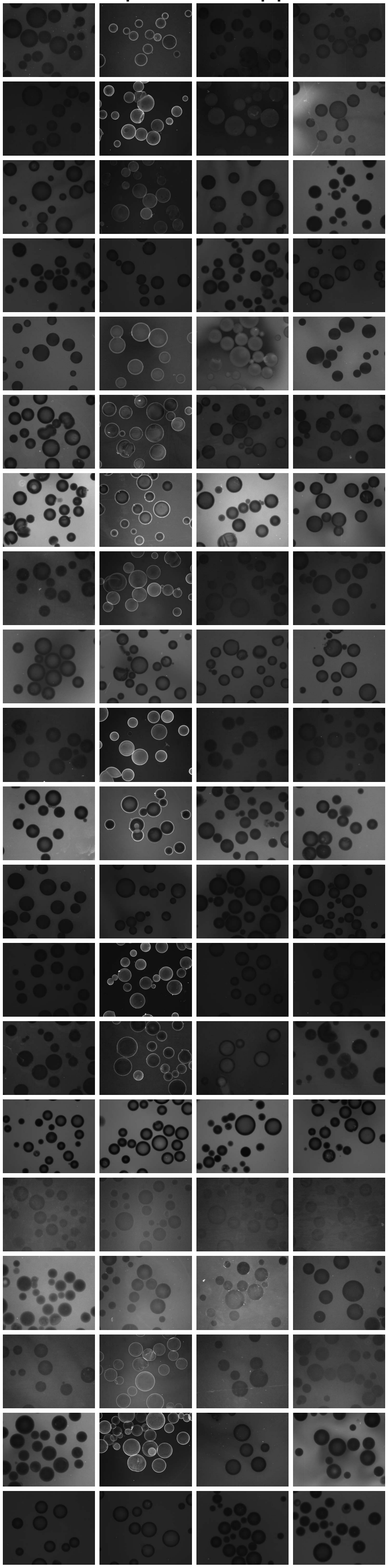

Bead Halo Assay (15/15)

Imp-α/β (4/4)

Rank by 2nd Z-score	Rank by 3rd Z-score	Accession	Gene Name	GST	GST-Imp-α2ΔN	GST-Imp-β	GST-Imp-β Q69L-Ran	Imp-α Direct Binding	Imp-β Direct Binding
1494	819	Q96IZ0	PAWR	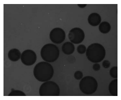	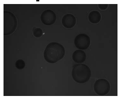	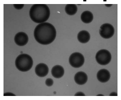	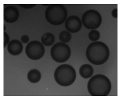	–	–
		Q9BTZ2	DHRS4	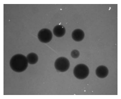	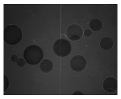	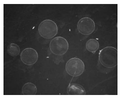	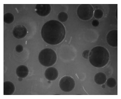	–	++

Western Blotting (1/2)

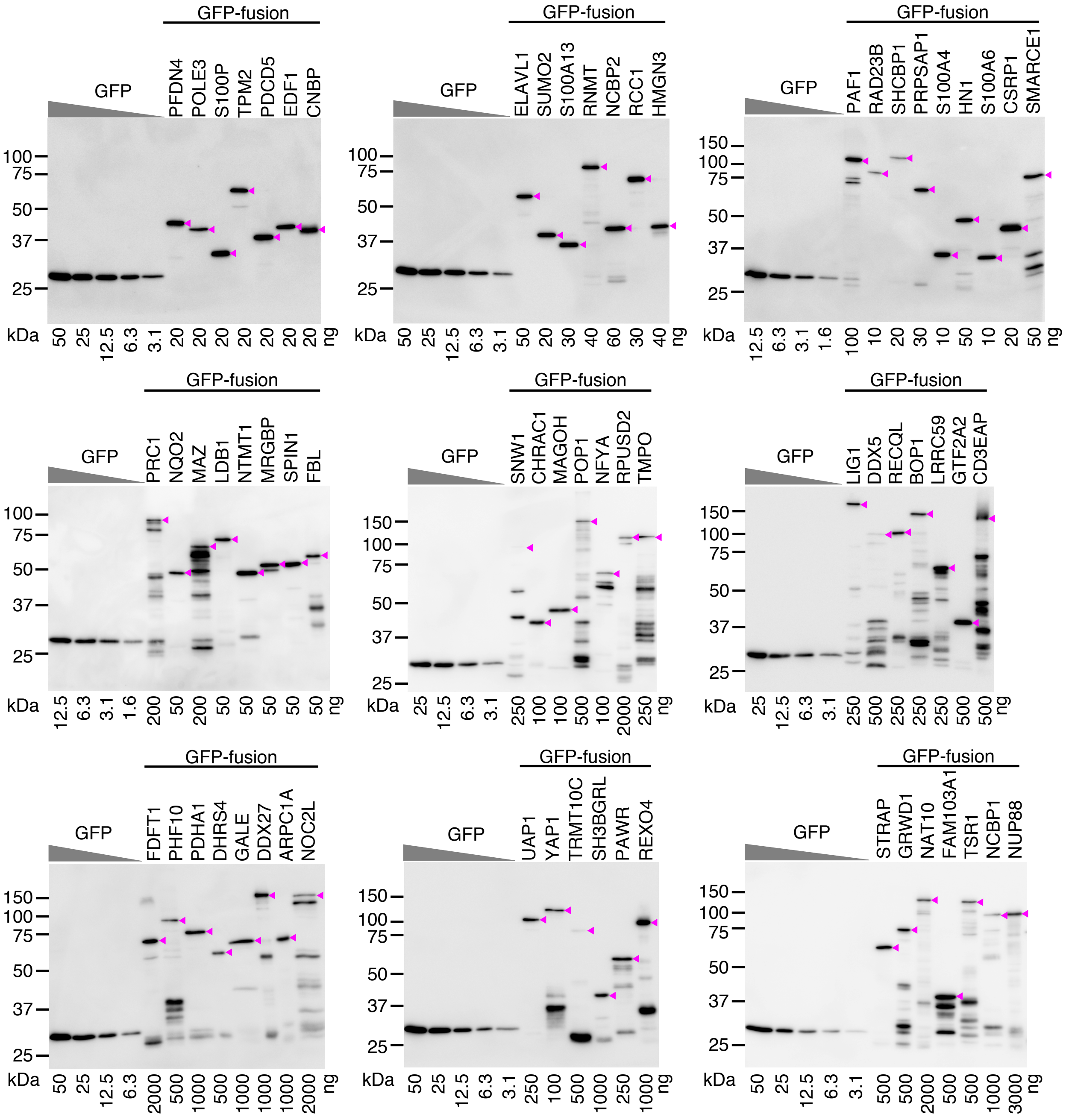

Western Blotting (2/2)

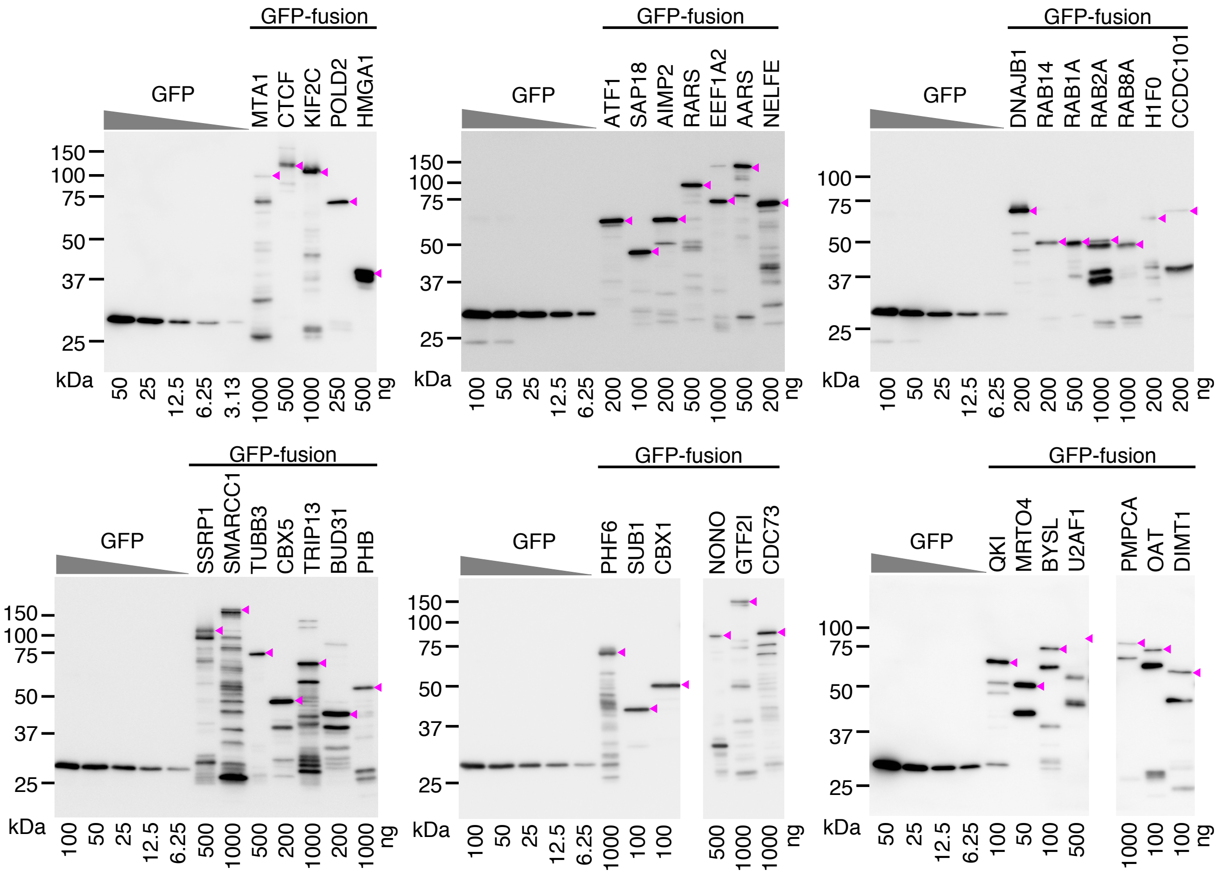

Gene Name	UniProt Accession	NCBI Protein	Deletion, Substitution, or Insertion in Used Protein
AARS	P49588	NP_001596.2	MDSTLTASEIRQRFIDFFKRNEHTYVHSSATIPLDDPTLLFANAGMNQFKPIFLNTIDPSHPMAKLSRAANTQKCIRAGGKHNDLDDVGKDVYHHTFFEMLGSWSFGDYFKELACKMALELLTQEFGIPIERLYVTYFGGDEAAGLEADLECKQIWQNLGLDDTKILPGNMKDNFWEMGDTGPCGPCSEIHYDRIGGRDAAHLVNQDDPNVLEIWNLVFIQYNREADGILKPLPKKSIDTGMGLERLVSVLQNKMSNYDTDLFVPYFEAIQKGTGARPYTGKVGAEDADGIDMAYRVLADHARTITVALADGGRPDNTGRGYVLRRILRRAVRYAHEKLNASRGFFATLVDVVVQSLGDAFPELKKDPDMVKDIINEEEVQFLKTLSRGRRILDRKIQSLGDSKTIPGDTAWLLYDTYGFPVDLTGLIAEEKGLVVDMDGFEEERKLAQLKSQGKGAGGEDLIMLDIYAIEELRARGLEVTDDSPKYNYHLDSSGSYVFENTVATVMALRREKMFVEEVSTGQECGVVLDKTCFYAEQGGQIYDEGYLVKVDDSSEDKTEFTVKNAQVRGGYVLHIGTIYGDLKVGDQVWLFIDEPRRRPIMSNHTATHILNFALRSVLGEADQKGSLVAPDRLRFDFTAKGAMSTQQIKKAEEIANEMIEAAKAVYTQDCPLAAAKAIQGLRAVFDETYPDPVRVVSIGVPVSELLDDPSGPAGSLTSVEFCGGTHLRNSSHAGAFVIVTEEAIAKGIRRIVAVTGAEAQKALRKAESLKKCLSVMEAKVKAQTAPNKDVQREIADLGEALATAVIPQWQKDELRETLKSLKKVMDDLDRASKADVQKRVLEKTKQFIDSNPNQPLVILEMESGASAKALNEALKLFKMHSPQTSAMLFTVDNEAGKITCLCQVPQNAANRGLKASEWVQQVSGLMDGKGGGKDVSAQATGKNVGCLQEALQLATSFAQLRLGDVKN
AIMP2	Q13155	NP_006294.2	
ARPC1A	Q92747	NP_006400.2	
ATF1	P18846	NP_005162.1	
BOP1	Q14137	NP_056016.1	MAGSRGAGRTAAPSVRPEKRRSEPELEPEPEPEPPLLCTSPLSHSTGSDSGVSDSEESVFSGLEDSGSDSSEDDDEGDEEGEDGALDDEGHSGIKKTTEEQVQASTPCPRTEMASARIGDEYAEDSSDEEDIRNTVGNVPLEWYDDFPHVGYDLDGRCIYKPLRTRDELDQFLDKMDDPDYWRTVQDPMTGRDLRLTDEQVALVRRLQSGQFGDVGFNPYEPAVDFFSGDVMIHPVTNRPADKRSFIPSLVEKEKVSRMVHAIKMGWIQPRRPRDPTPSFYDLWAQEDPNAVLGRHKMHVPAPKLALPGHAESYNPPPEYLLSEEERLAWEQQEPGERKLSFLPRKFPSLRAVPAYGRFIQERFERCLDLYLCPRQRKMRVNVDPEDLIPKLPRPRDLQPFPTCQALVYRGHSDLVRCLSVSPGGQWLVSGSDDGSLRLWEVATARCVRTVPVGGVVKSVAWNPSPAVCLVAAAVEDSVLLLNPALGDRLVAGSTDQLLSAFVPPEEPPLQPARWLEASEEERQVGLRLRICHGKPVTQVTWHGRGDYLAVVLATQGHTQVLIHQLSRRRSQSPFRRSHGQVQRVAFHPARPFLLVASQRSVRLYHLLRQELTKKLMPNCKWVSSLAVHPAGDNVICGSYDSKLVWFDLDLSTKPYRMLRHHKKALRAVAFHPRYPLFASGSDDGSVIVCHGMVYNDLLQNPLLVPVKVLKGHVLTRDLGVLDVIFHPTQPWVFSSGADGTVRLFT
BUD31	P41223	NP_003901.2	
BYSL	Q13895	NP_004044.3	
CBX1	P83916	NP_006798.1	
CBX5	P45973	NP_001120794.1	
CCDC101	Q96ES7	NP_612423.1	
CD3EAP	O15446	NP_001284519.1	MEEPQAGGEDAARFSCPPNFTAKPPASESPRFSLEALTGPDTELWLIQAPADFAPECFNGRHVPLSGSQIVKGKLAGKRHRYRVLSSCPQAGEATLLAPSTEAGGGLTCASAPQGTLRILEGPQQSLSGSPLQPIPASPPPQIPPGLRPRFCAFGGNPPVTGPRSALAPNLLTSGKKKKEMQVTEAPVTQEAVNGHGALEVDMALGSPEMDVRKKKKKKNQQLKEPEAAGPVGTEPTVETLEPLGVLFPSTTKKRKKPKGKETFEPEDKTVKQEQINTEPLEDTVLSPTKKRKRQKGTEGMEPEEGVTVESQPQVKVEPLEEAIPLPPTKKRKKEKGQMAMMEPGTEAMEPVEPEMKPLESPGGTMAPQQPEGAEPQAQAALAAPKKKTKKEKQQNATVEPETEVVGPELPDDLEPQAAPTSTKKKKKKKERGHTVTEPIQPLEPELPGEGQPEARATPGSTKKRKKQSQESRMPETVPQEEMPGPPLNSESGEEAPTGRDKKRKQQQQQPV
CDC73	Q6P1J9	NP_078805.3	
CHRAC1	Q9NRG0	NP_059140.1	
CNBP	P62633	NP_003409.1	
CSRP1	P21291	NP_004069.1	
CTCF	P49711	NP_006556.1	
DDX27	Q96GQ7	NP_060365.7	MVLAQRRRGGCEKLRAGPQAVLASGSGFCDDMLADLGLIGTIGEDDEVPVEPESDSGDEEEEGPIVLGRRQKALGKNRSADFNPDFVFTEKEGTYDGSWALADVMSQLKKKRAATTLDEKIEKVRKKRKTEDKEAKSGKLEKEKEAKEGSEPKEQEDLQENDEEGSEDEASETDYSSADENILTKADTLKVKDRKKKKKKGQEAGGFFEDASQYDENLSFQDMNLSRPLLKAITAMGFKQPTPIQKACIPVGLLGKDICACAATGTGKTAAFALPVLERLIYKPRQAPVTRVLVLVPTRELGIQVHSVTRQLAQFCNITTCLAVGGLDVKSQEAALRAAPDILIATPGRLIDHLHNCPSFHLSSIEVLILDEADRMLDEYFEEQMKEIIRMCSHHRQTMLFSATMTDEVKDLASVSLKNPVRIFVNSNTDVAPFLRQEFIRIRPNREGDREAIVAALLTRTFTDHVMLFTQTKKQAHRMHILLGLMGLQVGELHGNLSQTQRLEALRRFKDEQIDILVATDVAARGLDIEGVKTVINFTMPNTIKHYVHRVGRTARAGRAGRSVSLVGEDERKMLKEIVKAAKAPVKARILPQDVILKFRDKIEKMEKDVYAVLQLEAEEKEMQQSEAQINTAKRLLEKGKEAVVQEPERSWFQTKEERKKEKIAKALQEFDLALRGKKKRKKFMKDAKKKGEMTAEERSQFEILKAQMFAERLAKRNRRAKRARAMPEEEPVRGPAKKQKQGKKSVFDEELTNTSKKALKQYRASPSFEEREQLGLPHQRRGGNFKSKSRYKRRK
DDX5	P17844	NP_004387.1	
DHRS4	Q9BTZ2	NP_066284.2	MHKAGLLGLCARAWNSVRMASSGMTHRDPLANKVALVTASTDGIGFAIARRLAQDGAHVVVSSRKQQNVDQAVATLQGEGLSVTGTVCHLGKAEDRERLVATAVKLHGGIDILVSNAAVNPFFGSIMDVTEEVWDKTLDINVKAPALMTKAVVPEMEKRGGGSVVIVSSIAAFSPSPGFSPYNVSKTALLGLTKTLAIELAPRNIRVNCLAPGLIKTSFSRMLWMDKEKEESMKETLRIRRLGEPEDCAGIVSFLCSEDASYITGETVVVGGGTPSRL
DIMT1	Q9UNQ2	NP_055288.1	
DNAJB1	P25685	NP_006136.1	
EDF1	O60869	NP_003783.1	
EEF1A2	Q05639	NP_001949.1	MGKEKTHINIVVIGHVDSGKSTTTGHLIYKCGGIDKRTIEKFEKEAAEMGKGSFKYAWVLDKLKAERERGITIDISLWKFETTKYYITIIDAPGHRDFIKNMITGTSQADCAVLIVAAGVGEFEAGISKNGQTREHALLAYTLGVKQLIVGVNKMDSTEPAYSEKRYDEIVKEVSAYIKKIGYNPATVPFVPISGWHGDNMLEPSPNMPWFKGWKVERKEGNASGVSLLEALDTILPPTRPTDKPLRLPLQDVYKIGGIGTVPVGRVETGILRPGMVVTFAPVNITTEVKSVEMHHEALIEALPGDNVGFNVKNVSVKDIRRGNVCGDSKSDPPQEAAQFTSQVIILNHPGQISAGYSPVIDCHTAHIACKFAELKEKIDRRSGKKLEDNPKSLKSGDAAIVEMVPGKPMCVESFSQYPPLGRFAVRDMRQTVAVGVIKNVEKKSGGAGKVTKSAQKAQKAGK
ELAVL1	Q15717	NP_001410.2	
FAM103A1	Q9BTL3	NP_113640.1	

Gene Name	UniProt Accession	NCBI Protein	Deletion, Substitution, or Insertion in Used Protein
FBL	P22087	NP_001427.2	
FDFT1	P37268	NP_001274679.1	MAAAACGTKAMALFKRTLVLSPAAAPRGPGAGTAPRGCCLPPAAWPCKDWPRGEGGRLWSRLPQSHSHSHSHSHSHSCSSTSPTAVCVVCPQDSLSSSLKTCYKYLNQ<br>TSRSFAAVIQALDGEMRNAVCIFYLVLRALDTLEDDMTISVEKKVPLLHNFHSFLYQPDWRFMESKEKDRQVLEDFPTISLEFRNLAEKYQTVIADICRRMGIGMAEFLDKHV<br>TSEQEWDKYCHYVAGLVGIGLSRLFSASEFEDPLVGEDTERANSMGLFLQKTNIIRDYLEDQQGGREFWPQEVWSRYVKKLGDFAKPENIDLAVQCLNELITNALHHIPDVI<br>TYLSRLRNQSVFNFCAIPQVMAIATLAACYNNQQVFKGAVKIRKGQAVTLMMDATNMPAVKAIIYQYMEEIYHRIPDSDPSSSKTRQIISTIRTQNLPNCQLISRSHYSPIYLSF<br>VMLLAALSWQYLTTLSQVTEDYVQTGEH
GALE	Q14376	NP_000394.2	
GRWD1	Q9BQ67	NP_113673.3	
GTF2A2	P52657	NP_004483.1	
GTF2I	P78347	NP_127492.1	MAQVAMSTLPVEDEESSESRMVVTFLMSALESMCKELAKSKAEVACIAVYETDVFVVGTERGRAFVNTRKDFQKDFVKYCVEEEEKAAEMHKMKSTTQANRMSVDAVEIE<br>TLRKTVEDYFCFCYGKALGKSTVVPVPYEKMLRDQSAVVVQGLPEGVAFKHPENYDLATLKWILENKAGISFIIKRPFLEPKKHVGGRVMVTDADRSILSPGGSCGPIKVKT<br>EPTEDSGISLEMAAVTVKEESEDPDYYQYNIQAGPSETDDVDEKQPLSKPLQGSHHSSEGNEGTEMEVPAEDSTQHVPSETSEDPEVEVTIEDDDYSPPSKRPKANELPQ<br>PPVPEPANAGKRKVREFNFEKWNARITDLRKQVEELFERKYAQAIKAKGPVTIPYPLFQSHVEDLYVEGLPEGIPFRRPSTYGIPRLERILLAKERIRFVIKKHELLNSTREDLQ<br>LDKPASGVKEEWYARITKLRKMVDQLFCKKFAEALGSTEAKAVPYQKFEAHPNDLYVEGLPENIPFRSPSWYGIPRLEKIIQVGNRIKFVIKRPELLTHSTTEVTQPRTNTPVK<br>EDWNVRITKLRKQVEEIFNLKFAQALGLTEAVKVPYPVFESNPEFLYVEGLPEGIPFRSPTWFGIPRLERIVRGSNKIKFVVKKPELVISYLPPGMASKINTKALQSPKRPRSP<br>GSNSKVPEIEVTVEGPNNNNPQTSAVRTPTQTNGSNVPFKPRGREFSFEAWNAKITDLKQKVENLFNEKCGEALGLKQAVKVPFALFESFPEDFYVEGLPEGVPFRRPSTF<br>GIPRLEKILRNKAKIKFIIKKPEMFETAIKESTSSKSPPRKINSSPNVNTTASGVEDLNIIQVTIPDDDNERLSKVEKARQLREQVNDLFSRKFGEAIGMGFPVKVPYRKITINPG<br>CVVVDGMPPGVSFKAPSYLEISSMRRILDSAEFIKFTVIRPFPGLVINNQLVDQSESEGPVIQESAEPSQLEVPATEEIKETDGSSQIKQEPDPTW
H1F0	P07305	NP_005309.1	
HMGA1	P17096	NP_665906.1	
HMGN3	Q15651	NP_004233.1	
HN1	Q9UK76	NP_057269.1	
KIF2C	Q99661	NP_006836.2	MAMDSSLQARLFPGLAIKIQRSNGLIHSANVRTVNLEKSCVSVEWAEGGATKGKEIDFDDVAAINPELLQLLPLHPKDNLPLQENVTIQKQKRRSVNSKIPAPKESLRSRSTR<br>MSIVSELRITAQENDMEVELPAAANSRKQFSVPPAPTRPSCPAVAEIPLRMVSEEMEEQVHSIRGSSSANPVNSVRRKSCLVKEVEKMKNKREEKKAQNSEMRMKRAQEY<br>DSSFPNWEFARMIKEFRATLECHPLTMTDPIEEHRICVCVRKRPLNKQELAKKEIDVISIPSKCLLLVHEPKLKVDLTKYLENQAFCFDFAFDETASNEVVYRFTARPLVQTIFE<br>GGKATCFAYGQTGSGKTHTMGGDLSGKAQNASKGIYAMASRDVFLLKNQPCYRKLGLEVYVTFFEIYNGKLFDLLNKKAKLRVLEDGKQQVQVVGLQEHLVNSADDVIKMI<br>DMGSACRTSGQTFANSNSSRSHACFQIILRAKGRMHGKFSLVDLAGNERGADTSSADRQTRMEGAEINKSLLALKECIRALGQNKAHTPFRESKLTQVLRDSFIGENSRTC<br>MIATISPGISSCEYTLNTLRYADRVKELSPHSGPSGEQLIQMETEEMEACSNGALIPGNLSKEEEELSSQMSSFNEAMTQIRELEEKAMEELKEIIQQGPDWLELSEMTEQP<br>DYDLETFVNKAESALAQQAKHFSALRDVIKALRLAMQLEEQASRQISSKKRPQ
LDB1	Q86U70	NP_001106878.1	MSVGCACPGCSSKSFKLYSPKEPPNGNAFPPFHPGTMLDRDVGPTPMYPPTYLEPGIGRHTPYGNQTDYRIFELNKRLQNWTEECDNLWWDAFTTEFFEDDAMLTITFCL<br>EDGPKRYTIGRTLIPRYFRSIFEGGATELYYVLKHPKEAFHSNFVSLDCDQGSMVTQHGKPMFTQVCVEGRLYLEFMFDDMMRIKTWHFSIRQHRELIPRSILAMHAQDPQ<br>MLDQLSKNITRCGLSNSTLNYLRLCVILEPMQELMSRHKTYSLSPRDCLKTCLFQKWQRMVAPPAEPTRQQPSKRRKRKMSGGSTMSSGGGNTNNSNSKKKSPASTFAL<br>SSQVPDVMVVGEPTLMGGEFGDEDERLITRLENTQFDAANGIDDEDSFNNSPALGANSPWNSKPPSSQESKSENPTSQASQ
LIG1	P18858	NP_000225.1	
LRRC59	Q96AG4	NP_060979.2	
MAGOH	P61326	NP_002361.1	
MAZ	P56270	NP_001036004.1	MFPVFPCTLLAPPFPVLGLDSRGVGGLMNSFPPPQGHAQNPLQVGAELQSRFFASQGCAQSPFQAAPAPPPTPQAPAAEPLQVDLLPVLAAAQESAAAAAAAAAAAAAVA<br>AAPPAPAAASTVDTAALKQPPAPPPPPPPVSAPAAEAAPPASAATIAAAAATAVVAPTSTVAVAPVASALEKKTKSKGPYICALCAKEFKNGYNLRRHEAIHTGAKAGRVPSGA<br>MKMPTMVPLSLLSVPQLSGAGGGGGEAGAGGGAAAVAAGGVVTTTASGKRIRKNHACEMCGKAFRDVYHLNRHKLSHSDEKPYQCPVCQQRFKRKDRMSYHVRSHDG<br>AVHKPYNCSHCGKSFSRPDHLNSHVRQVHSTERPFKCEKCEAAFATKDRLRAHTVRHEEKVPCHVCGKMLSSAYISDHMKVHSQGPHHVCELCNKGFTTAAYLRIHAVKD<br>HGLQAPRADRILCKLCSVHCKTPAQLAGHMQTHLGGAAPPVPGDAPQPQPTC
MRGBP	Q9NV56	NP_060740.1	
MRTO4	Q9UKD2	NP_057267.2	
MTA1	Q13330	NP_004680.2	MAANMYRVGDYVYFENSSSNPYLIRRIEELNKTANGNVEAKVVCFYRRRDISSTLIALADKHARLSVCYKAGPGADNGEEGEIEEEMENPEMVDLPEKLKHQLRHRELFLS<br>RQLESLPATHIRGKCSVTLLNETESLKSYLEREDFFFYSLVYDPQQKTLLADKGEIRVGNRYQADITDLLKEGEEDGRDQSRLETQVWEAHNPLTDKQIDQFLVVARSVGTF<br>ARALDCSSSVRQPSLHMSAAAASRDITLFHAMDTLHKNIYDISKAISALVPQGGPVLCRDEMEEWSASEANLFEEALEKYGKDFTDIQQDFLPWKSLTSIIEYYYMWKTTDR<br>YVQQKRLKAAEAESKLKQVYIPNYNKPNPNQISVNNVKAGVVNGTGAPGQSPGAGRACESCYTTQSYQWYSWGPPNMQCRLCASCWTYWKKYGGLKMPTRLDGERP<br>GPNRSNMSPHGLPARSSGSPKFAMKTRQAFYLHTTKLTRIARRLCREILRPWHAARHPYLPINSAAIKAECTARLPEASQSPLVLKQAVRKPLEAVLRYLETHPRPPKPDPV<br>KSVSSVLSSLTPAKVAPVINNGSPTILGKRSYEQHNGVDGNMKKRLLMPSRGLANHGQARHMGPSRNLLLNGKSYPTKVRLIRGGSLPPVKRRRMNWIDAPDDVFYMATE<br>ETRKIRKLLSSSETKRAARRPYKPIALRQSQALPPRPPPPAPVNDEPIVIED

Gene Name	UniProt Accession	NCBI Protein	Deletion, Substitution, or Insertion in Used Protein
NAT10	Q9H0A0	XP_005253056.1	MHRKKVDNRIRILIENGVAERQRSLFVVVGDRGKDQVVILHHMLSKATVKARPSVLWCYKKELGFSSHRKKRMRQLQKKIKNGTLNIKQDDPFELFIAATNIRYCYYNETHKILGNTFGMCVLQDFEALTPNLLARTVETVEGGGLVVILLRTMNSLKQLYTVTMDVHSRYRTEAHQDVVGRFNERFILSLASCKKCLVIDDQLNILPISSHVATMEALPPQTPDESLGPSDLELRELKESLQDTQPVGVLVDCCKTLDQAKAVLKFIEGISEKTLRSTVALTAARGRGKSAALGLAIAGAVAFGYSNIFVTSPSPDNLHTLFEFVFKGFDALQYQEHLDYEIIQSLNPEFNKAVIRVNVFREHRQTIQYIHPADAVKLGQAELVVIDEAAAIPLPLVKSLLGPYLVFMASTINGYEGTGRSLSLKLIQQLRQQSAQSQVSTTAENKTTTTARLASARTLHEVSLQESIRYAPGDAVEKWLNDLLCLDCLNITRIVSGCPLPEACELYYVNRDTLFCYHKASEVFLQRLMALYVASHYKNSPNDLQMLSDAPAHHLFCLLPPVPPTQNALPEVLAVIQVCLEGEISRQSILNSLSRGKKASGDLIPWTVSEQFQDPDFGGLSGGRVVRIAVHPDYQGMGYGSRALQLLQMYYEGRFPCLEEKVLETPQEIHTVSSEAVSLLEEVITPRKDLPPLLLKLNERPAERLDYLGVSYGLTPRLLKFWKRAGFVPVYLRQTPNDLTGEHSCIMLKTLTDEDEADQGGWLAAFWKDFRRRFLALLSYQFSTFSPSLALNIIQNRNMGKPAQPALSREELEALFLPYDLKRLEMYSRNMVDYHLIMDMIPAISRIYFLNQLGDLALSAAQSALLLGIGLQHKSVDQLEKEIELPSGQLMGLFNRIIRKVVKLFNEVQEKAIEEQMVAAKDVVMEPTMKTLSDDLDEAAKEFQEKHKKEVGKLKSMDLSEYIIRGDDEEWNEVLNKAGPNASIISLKSDKKRKLEAKQEPKQSKKLKNRETKNKKDMKLKRKK
NCBP1	Q09161	NP_002477.1	MSRRRHSDENDGGQPHKRRKTSDANETEDHLESLICKVGEKSACSLESNLEGLAGVLEADLPNYKSKILRLLCTVARLLPEKLTIYTTLVGLLNARNYNFGGEFVEAMIRQLKESLKANNYNEAVYLVRFLSDLVNCHVIAAPSMVAMFENFVSVTQEEDVPQVRRDWYVYAFLSSLPWVGKELYEKKDAEMDRIFANTESYLKRRQKTHVPMLQVWTADKPHPQEEYLDCLWAQIQKLKKDRWQERHILRPYLAFDSILCEALQHNLPPFTPPPHTEDSVYPMPRVIFRMFDYTDDPEGPVMPGSHSVERFVIEENLHCIIKSHWKERKTCAAQLVSYPGKNKIPLNYHIVEVIFAELFQLPAPPHIDVMYTTLLIELCKLQPGSLPQVLAQATEMLYMRLDTMNTTCVDRFINWFSHHLSNFQFRWSWEDWSDCLSQDPESPKPKFVREVLEKCMRLSYYQRILDIVPPTFSALCPANPTCIYKYGDESSNSLPGHSVALCLAVAFKSKATNDEIFSILKDVPNPNQDDDDDEGFSFNPLKIEVFVQTLLHLAAKSFSHSFSALAKFHEVFKTLAESDEGKLHVLRVMFEVWRNHPQMIAVLVDKMIRTQIVDCAAVANWIFSSELSRDFTRLFVWEILHSTIRKMNKHVLKIQKELEEAKEKLARQHKRRSDDDDRSSDRKDGALEEQIERLQEKVESAQSGQKNLFLVIFQRFIMILTEHLVRCETDGTSVLTPWYKNCIERLQQIFLQHHQIIQQYMVTLENLLFTAELDPHILAVFQQFCALQA
NCBP2	P52298	NP_031388.2	
NELFE	P18615	NP_002895.3	
NFYA	P23511	NP_002496.1	MEQYTANSNSSTEQIVVQAGQIQQQQQGGVTAVQLQTEAQVASASGQQVQTLQVVQGQPLMVQVSGGQLITSTGQPIMVQAVPGGQGQTIMQVPVSGTQGLQQIQLVPPGQIQIQGGQAVQVQGQQGQTQQIIIQQPQTAVTAGQTQTQQQIAVQGQQVAQTAEGQTIVYQPVNADGTILQQVTVPVSGMITIPAASLAGAQIVQTGANTNTTSSGQGTVTVTLPVAGNVVNSGGMVMMVPGAGSVPAIQRIPLPGAEMLEEEPLYVNAKQYHRILKRRQARAKLEAEGKIPKERRKYLHESRHRHAMARKRGEGGRFFSPKEKDSPHMQDPNQADEEAMTQIIRVS
NOC2L	Q9Y3T9	NP_056473.2	MAAAGSRKRRLAELTVDEFLASGFDSESESESENSPQAETREAREAARSPDKPGGSPSASRRKGRASEHKDQLSRLKDRDPEFYKFLQENDQSLLNFSDSDSSEEEEGPFHSLPDVLEEASEEEDGAEEGEDGDRVPRGLKGKKNSVPVTVAMVERWKQAAKQRLTPKLFHEVVQAFRAAVATTRGDQESAEANKFQVTDSAAFNALVTFCIRDLIGCLQKLLFGKVAKDSSRMLQPSSSPLWGKLRVDIKAYLGSAIQLVSCLSETTVLAAVLRHISVLVPCFLTFPKQCRMLLKRMVVVWSTGEESLRVLAFLVLSRVCRHKKDTFLGPVLKQMYITYVRNCKFTSPGALPFISFMQWTLTELLALEPGVAYQHAFLYIRQLAIHLRNAMTTRKKETYQSMYNWQYVHCLFLWCRVLSTAGPSEALQPLVYPLAQVIIGCIKLIPTARFYPLRMHCIRALTLLSGSSGAFIPVLPFILEMFQQVDFNRKPGRMSSKPINFSVILKLSNVNLQEKAYRDGLVEQLYDLTLEYLHSQAHCIGFPELVLPVVLQLKSFLRECKVANYCRQVQQLLGKVQENSAYICSRRQRVSFGVSEQQAVEAWEKLTREEGTPLTLYYSHWRKLRDREIQLEISGKERLEDLNFPEIKRRKMADRKDEDRKQFKDLFDLNSSEEDDTEGFSERGILRPLSTRHGVEDDEEDEEEGEEDSSNSEGEWSWDGDPDAEAGLAPGELQQLAQGPEDELEDLQLSEDD
NONO	Q15233	NP_031389.3	
NQO2	P16083	NP_001277150.1	MAGKKVLIVYAHQEPRSFNGSLKNVAVDELSRQGCTVTVSDLYAMNFEPRATDKDITGTLSNPEVFNYGVETHEAYKQRSLASDITDEQKKVREADLVIFQFPLYWFSVPAILKGWMDRVLCQGFAFDIPGFYDSGLLQGKLALLSVTTGGTAEMYTKTGVNGDSRYFLWPLQHGTLHFCGFKVLAPQISFAPEIASEEERKGMVAAWSQRLQTIWKEEPIPCTAHWHFGQ
NTMT1	Q9BV86	NP_001273725.1	
NUP88	Q99567	NP_002523.2	MEAAEGPVGDGELWQTWLPNHVVFLRLREGLKNQSPTEAEKPASSSLPSSPPPQLLTRNVVFGLGGELFLWDGEDSSFLVVRLRGPSGGGEELALSQYQRLLCINPPLFEIYQVLLSPTQHHVALIGIKGLMVLELPKRWGKNSEFEGGKSTVNCSTTPVAERFFTSSTSLTLKHAAWYPSEILDPHVVLLTSDNVIRIYSLREPQTPTNVIILSEAEEESLVLNKGRAYTASLGETAVAFDFGPLAAVPKTLFGQNGKDEVVAYPLYILYENGETFLTYISLLHSPGNIGKLLGPLPMHPAAEDNYGYDACAVLCLPCVPNILVIATESGMLYHCVVLEGEEEDDHTSEKSWDSRIDLIPSLYVFECVELELALKLASGEDDPFDSDFSCPVKLHRDPKCPSRYHCTHEAGVHSVGLTWIHKLHKFLGSDEEDKDSLQELSTEQKCFVEHILCTKPLPCRQPAPIRGFWIVPDILGPTMICITSTYECLIWPLLSTVHPASPPLLCTREDVEVAESPLRVLAETPDSFEKHIRSILQRSVANPAFLKASEKDIAPPPEECLQLLSRATQVFREQYILKQDLAKEEIQRRVKLLCDQKKKQLEDLSYCREERKSLREMAERLADKYEEAKEKQEDIMNRMKKLLHSFHSELPVLSDSERDMKKELQLIPDQLRHLGNAIKQVTMKKDYQQQKMEKVLSLPKPTIILSAYQRKCIQSILKEEGEHIREMVKQINDIRNHVNF
OAT	P04181	NP_000265.1	
PAF1	Q8N7H5	NP_061961.2	
PAWR	Q96IZ0	NP_002574.2	MATGGYRTSSGLGGSTTDFLEEWKAKREKMRAKQNPPGPAPPGGGSSDAAGKPPAGALGTPAAAAANELNNNLPGGAPAAPAVPGPGGVNCAVGSAMLTRAAPGPRRSEDEPPAASASAAPPPQRDEEEPDGVPEKGKSSGPSARKGKGQIEKRKLREKRRSTGVVNIPAAECLDEYEDDEAGQKERKREDAITQQNTIQNEAVNLLDPGSSYLLQEPPRTVSGRYKSTTSVSEEDVSSRYSRTDRSGFPRYNRDANVSGTLVSSSTLEKKIEDLEKEVVRERQENLRLVRLMQDKEEMIGKLKEEIDLLNRDLDDIEDENEQLKQENKTLLKVVGQLTR
PDCD5	O14737	NP_004699.1	
PDHA1	P08559	NP_001166925.1	MRKMLAAVSRVLSGASQKPRHGLATLPSLVSISRLKQSSHLGLPKCWDYSHSLKTRQASRVLVASRNFANDATFEIKKCDLHRLEEGPPVTTVLTREDGLKYYRMMQTVRRMELKADQLYKQKIIRGFCHLCDGQEACCVGLEAGINPTDHLITAYRAHGFTFTRGLSVREILAELTGRKGGCAKGKGGSMHMYAKNFYGGNGIVGAQVPLGAGIALACKYNGKDEVCLTLYGDGAANQGQIFEAYNMAALWKLPCIFICENNRYGMGTSVERAAASTDYYKRGDFIPGLRVDGMDILCVREATRFAAAYCRSGKGPILMELQTYRYHGHSMSDPGVSYRTREEIQEVRSKSDPIMLLKDRMVNSNLASVEELKEIDVEVRKEIEDAAQFATADPEPPLEELGYHIYSSDPPFEVRGANQWIKFKSVS
PFDN4	Q9NQP4	NP_002614.2	
PHB	Q99623	NP_002625.1	
PHF10	Q8WUB8	NP_060758.2	
PHF6	Q8IWS0	NP_115834.1	
PMPCA	Q10713	NP_055975.1	

Gene Name	UniProt Accession	NCBI Protein	Deletion, Substitution, or Insertion in Used Protein
POLD2	P49005	NP_001120690.1	
POLE3	Q9NRF9	NP_001265184.1	
POP1	Q99575	NP_001139332.1	
PRC1	O43663	NP_003972.1	
PRPSAP1	Q14558	NP_002757.2	
QKI	Q96PU8	NP_006766.1	
RAB14	P61106	NP_057406.2	
RAB1A	P62820	NP_004152.1	
RAB2A	P61019	NP_002856.1	
RAB8A	P61006	NP_005361.2	
RAD23B	P54727	NP_002865.1	
RARS	P54136	NP_002878.2	
RCC1	P18754	NP_001041659.1	MSPKRIAKRRSPPADAIPKSKKVKDTRAAASRRVPGARSCQGACGPSPPDQKTRPVSHRSHSTEPGLVLTLGQGDVGQLGLGENVMERKKPALVSIPEDVVQAEAGGMHTVCLSKSGQVYSFGCNDEGALGRDTSVEGSEMVPGKVELQEKVVQVSAGDSHTAALTDDGRVFLWGSFRDNNGVIGLLEPMKKSMVPVQVQLDVPVVKVASGNDHLVMLTADGDLYTLGCGEQGQLGRVPELFANRGGRQGLERLLVPKCVMLKSRGSRGHVRFQDAFCGAYFTFAISHEGHVYGFGLSNYHQLGTPGTESCFIPQNLTSFKNSTKSWVGFSGGQHHTVCMDSEGKAYSLGRAEYGRLGLGEGAEEKSIPTLISRLPAVSSVACGASVGYAVTKDGRVFAWGMGTNYQLGTGQDEDAWSPVEMMGKQLENRVVLSVSSGGQHTVLLVKDKEQS
RECQL	P46063	NP_116559.1	
REXO4	Q9GZR2	NP_065118.2	
RNMT	O43148	NP_003790.1	
RPUSD2	Q8IZ73	NP_689473.1	
S100A13	Q99584	NP_001019381.1	
S100A4	P26447	NP_002952.1	
S100A6	P06703	NP_055439.1	
S100P	P25815	NP_005971.1	
SAP18	O00422	NP_005861.2	
SH3BGRL	O75368	NP_003013.1	
SHCBP1	Q8NEM2	NP_079021.3	MADGSLTGGGLEAAAMAPERTGWAVEQELASLEKGLFQDEDSCSDCSYRDKPGSSLQSFMPEGKTFFPEIFQTNQLLFYERFRAYQDYILADCKASEVQEFTAEFLEKVLEPSGWRAVWHTNVFKVLVEITDVDFAALKAVVRLAEPYLCDSQVSTFTMECMKELLDLKEHRLPLQELWVVFDDSGVFDQTALAIEHVRFFYQNIWRSWDEEEEDEYDYFVRCVEPRLRLHYDVLEDRVPSGLIVDYHNLLSQCEESYRKFLNLRSSLSNCNSDSEQENISMVEGLKLYSEMEQLKQKLKLIENPLLRYVFGYQKNSNIQAKGVRSSGQKITHVVSSTMMAGLLRSLLTDRLCQEPGEEEREIQFHSDPLSAINACFEGDTVIVCPGHYVVHGTFSIADSIELEGYGLPDDIVIEKRGKGDTFVDCTGADIKISGIKFVQHDAVEGILIVHRGKTTLENCVLQCETTGVTVRTSAEFLMKNSDLYGAKGAGIEIYPGSQCTLSDNGIHHCKEGILIKDFLDEHYDIPKISMVNNIIHNNEGYGVVLVKPTIFSDLQENAEDGTEENKALKIQTSGEPDVAERVDLEELIECATGKMELCARTDPSEQVEGNCEIVNELIAASTQKGQIKKKRLSELGITQADDNLMSQEVFVGIVGNQFKWNGKGSFGTFLF
SMARCC1	Q92922	NP_003065.3	MAAAAGGGGPGTAVGATGSGIAAAAAGLAVYRRKDGGPATKFWESPETVSQLDSVRVWLGKHYKKYVHADAPTNKTLAGLVVQLLQFQEDAFGKHVTNPAFTKLPAKCFMDFKAGGALCHILGAAYKYKNEQGWRRFDLQNPSRMDRNVEMFMNIEKTLVQNNCLTRPNIYLIPDIDLKLANKLKDIIKRHQGTFTDEKSKASHHIYPYSSSQDDEEWLRPVMRKEKQVLVHWGFYPDSYDTWVHSNDVDAEIEDPPIPEKPWKVHVKWILDTDIFNEWMNEEDYEVDENRKPVSFRQRISTKNEEPARSPERRDRKASANARKRKHSPSPPPPTPTESRKKSGKKGQASLYGKRRSQKEEDEQEDLTKDMEDPTPVPNIEEVVLPKNVNLKKDSENTPVKGGTVADLDEQDEETVTAGGKEDEDPAKGDQSRSVDLGEDNVTEQTNHIIIPSYASWFDYNCIHVIERRALPEFFNGKNKSKTPEIYLAYRNFMIDTYRLNPQEYLTSTACRRNLTGDVCAVMRVHAFLEQWGLVNYQVDPESRPMAMGPPPTPHFNVLADTPSGLVPLHLRSPQVPAAQQMLNFPEKNKEKPVDLQNFGLRTDIYSKKTLAKSKGASAGREWTEQETLLLLEALEMYKDDWNKVSEHVGSRTQDECILHFLRLPIEDPYLENSDASLGPLAYQPVPFSQSGNPVMSTVAFLASVVDPRVASAAAKAALEEFSRVREEVPLELVEAHVKKVQEAARASGKVDPTYGLESSCIAGTGPDEPEKLEGAEEEKMEADPDGQQPEKAENKVENETDEGDKAQDGENEKNSEKEQDSEVSEDTKSEEKETEENKELTDTCKERESDTGKKKVEHEISEGNVATAAAAALASAATKAKHLAAVEERKIKSLVALLVETQMKKLEIKLRHFEELETIMDREKEALEQQRQQLLTERQNFHMEQLKYAELRARQQMEQQQHGQNPQQAHQHSGGPGLAPLGAAGHPGMMPHQQPPPYPLMHHQMPPPHPPQPGQIPGPGSMMPGQHMPGRMIPTVAANIHPSGSGPTPPGMPPMPGNILGPRVPLTAPNGMYPPPPQQQPPPPPPADGVPPPPAPGPPASAAP
SMARCE1	Q969G3	NP_003070.3	
SNW1	Q13573	NP_036377.1	
SPIN1	Q9Y657	NP_006708.2	
SSRP1	Q08945	NP_003137.1	
STRAP	Q9Y3F4	NP_009109.3	
SUB1	P53999	NP_006704.3	
SUMO2	P61956	NP_008868.3	

Gene Name	UniProt Accession	NCBI Protein	Deletion, Substitution, or Insertion in Used Protein
TMPO	P42166	NP_003267.1	MPEFLEDPSVLTKDKLKSELVANNVTLPAGEQRKDVYVQLYLQHLTARNRPPLPAGTNSKGPPDFSSDEEREPTPVLGSGAAAAGRSRAAVGRKATKKTDKPRQEDKDDL<br>DVTELTNEDLLDQLVKYGVNPGPIVGTTRKLYEKKLLKLREQGTESRSSTPLPTISSSAENTRQNGSNDSDRYSDNEEGKKKEHKKVKSTRDIVPFSELGTTPSGGGFFQGI<br>SFPEISTRPPLGSTERQAAKKVHTSKGDLPREPLVATNLPGRGQLQKLASERNLFISCKSSHDRCLEKSSASSSQPEHSAMLVSTAASPSLIKETTTGYYKDIVENICGREKS<br>GIQPLCPERSHISDQSPLSSKRKALEESESSQLISPPLAQAIRDYVNSLLVQGGVGSLPGTSNSMPPLDVENIQKRIDQSKFQETEFLSPPRKVPRLSEKSVEERDSGSFVAF<br>QNIPGSELMSSFAKTVVSHSLTTLGLEVAKQSQHDKIDASELSFPFHESILKVIEEEWQQVDRQLPSLACKYPVSSREATQILSVPKVDDEILGFISEATPLGGIQAASTESCN<br>QQLDLALCRAYEAAASALQIATHTAFVAKAMQADISQAAQILSSDPSRTHQALGILSKTYDAASYICEAAFDEVKMAAHTMGNATVGRRYLWLKDCKINLASKNKLASTPFKG<br>GTLFGGEVCKVIKKRGNKH
TPM2	P07951	NP_003280.2	MDAIKKKMQMLKLDKENAIDRAEQAEADKKQAEDRCKQLEEEQQALQKKLKGTEDEVEKYSESVKEAQEKLEQAEKKATDAEADVASLNRRIQLVEEELDRAQERLATALQ<br>KLEEAEKAADESERGMKVIENRAMKDEEKMELQEMQLKEAKHIAEDSDRKYEEVARKLVILEGELERSEERAEVAESRARQLEEELRTMDQALKSLMASEEEYSTKEDKYE<br>EEIKLLEEKLKEAETRAEFAERSVAKLEKTIDDLEDEVYAQKMKYKAISEELDNALNDITSL
TRIP13	Q15645	NP_004228.1	
TRMT10C	Q7L0Y3	NP_060289.2	
TSR1	Q2NL82	NP_060598.3	
TUBB3	Q13509	NP_006077.2	
U2AF1	Q01081	NP_006749.1	
UAP1	Q16222	NP_003106.3	
YAP1	P46937	NP_001123617.1	
