# Supplementary material for: Extensive cargo identification reveals distinct biological roles of the 12 importin pathways
Source: eLife. 2017 Jan 24;6:e21184. doi: 10.7554/eLife.21184 (PMC5305215; doi:10.7554/eLife.21184)

Supplementary file 4

Example of extracted ion chromatograms (EICs) of peptides:
EICs of GLUD1 peptides in the SILAC-Tp with Trn-1

As a general problem of high-throughput LC-MS/MS quantitation, quantitative values of proteins with fewer quantified peptides deviate among the replicates. Referring to EICs of the quantified peptides is useful to avoid misidentification of cargoes. For example, the L/H ratios of a Trn-1 2nd-Z-15% cargo GLUD1 (P00367, ranked 110th and 342nd by the 2nd and 3rd Z-score, respectively) deviated largely in the three replicates of SILAC-Tp with Trn-1 (Supplementary file 1). Panels (A–H) show EICs of the indicated peptide (trypsin targets, K and R, are written in lower cases) in the three (three Ctl and there +Trn) experiments (some peptides were not identified in all the experiments). Magenta letters indicate the quantified peptides and L/H rations.

In panel (A), the elution time of the peptide TAMkYNLGLDLr differs largely between the expriment-1 Ctl and experiment-2 +Trn-1, the peak shape of the experiment-2 +Trn-1 is irregular, and the L/H ratio of it is much higher than those of other peptides in +Trn-1 experiments (B and E). Thus, there is concern about misidentification. Because the L/H count of GLUD1 in the experiment-2 +Trn-1 is two (TAMkYNLGLDLr and NLNHVSYGr, A and B) and the L/H ratio of a protein is defined as the median, the L/H ratio of GLUD1 in the expriment-2 +Trn-1 is affected by the L/H ratio of TAMkYNLGLDLr. Exclusion of the L/H ratio of TAMkYNLGLDLr in the experiment-2 +Trn-1 lowers the Z-score rank of GLUD1 significantly.

In panel (C), the chromatogram of the peptide HGGTIPIVPTAEFQDr in the experiment-1 Ctl has an irregular peak, and the L/H ratio of it is much higher than those of other peptides in Ctl experiments (A–H). Thus, overlap with other peptide or other failures may be possible. However, the L/H count of GLUD1 in the experiment-1 Ctl is four (TAMkYNLGLDLr, HGGTIPIVPTAEFQDr, ALASLMTYk, and GASIVEDkLVEDLr) and the value of HGGTIPIVPTAEFQDr does not affect the median. (The L/H ratio of TAMkYNLGLDLr, whose EIC differ between the experiment-1 Ctl and expriment-2 +Trn in (A) as mentioned above, may affect the L/H ratio of GLUD1 in the experiment-1 Ctl, but we assumed that it is reliable.)

As above, the L/H ratios of proteins with low L/H counts (Supplementary file 1) may be affected by LC-MS/MS artifacts, and misidentification can be avoided by referring to the EICs. All the EICs and MS spectra in this work can be accessed by downloading the mass spectrometry data and Proteome Discoverer software (see the Materials and Methods and Supplementary file 12A).

A TAMkYNLGLDLr

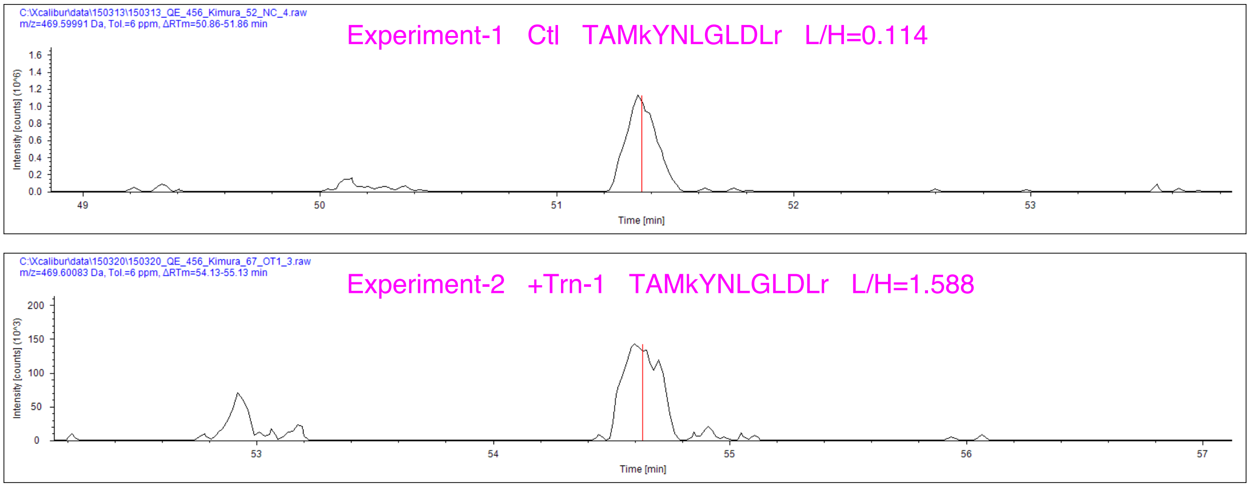

B NLNHVSYGr

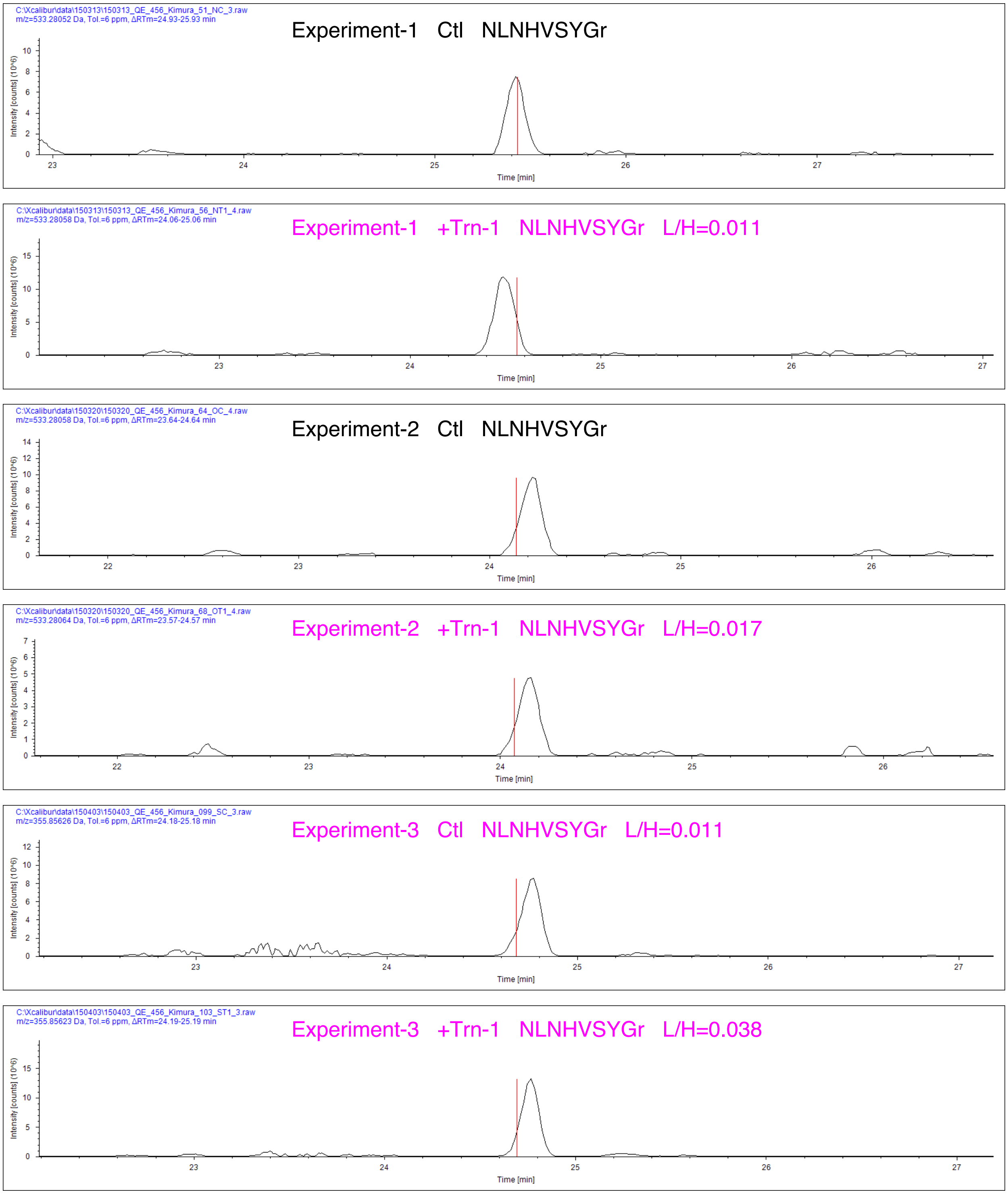
C:\Xcalibur\data\150313\150313_QE_456_Kimura_51_NC_3.raw
m/z=533.28052 Da, Tol.=6 ppm, ΔRTm=24.93-25.93 min
Experiment-1 Ctl NLNHVSYGr
Intensity [counts] (10^6)
Time [min]
C:\Xcalibur\data\150313\150313_QE_456_Kimura_56_NT1_4.raw
m/z=533.28058 Da, Tol.=6 ppm, ΔRTm=24.06-25.06 min
Experiment-1 +Trn-1 NLNHVSYGr L/H=0.011
C:\Xcalibur\data\150320\150320_QE_456_Kimura_64_OC_4.raw
m/z=533.28058 Da, Tol.=6 ppm, ΔRTm=23.64-24.64 min
Experiment-2 Ctl NLNHVSYGr
C:\Xcalibur\data\150320\150320_QE_456_Kimura_68_OT1_4.raw
m/z=533.28064 Da, Tol.=6 ppm, ΔRTm=23.57-24.57 min
Experiment-2 +Trn-1 NLNHVSYGr L/H=0.017
C:\Xcalibur\data\150403\150403_QE_456_Kimura_099_SC_3.raw
m/z=355.85626 Da, Tol.=6 ppm, ΔRTm=24.18-25.18 min
Experiment-3 Ctl NLNHVSYGr L/H=0.011
C:\Xcalibur\data\150403\150403_QE_456_Kimura_103_ST1_3.raw
m/z=355.85623 Da, Tol.=6 ppm, ΔRTm=24.19-25.19 min
Experiment-3 +Trn-1 NLNHVSYGr L/H=0.038

C HGGTIPIVPTAEFQDr

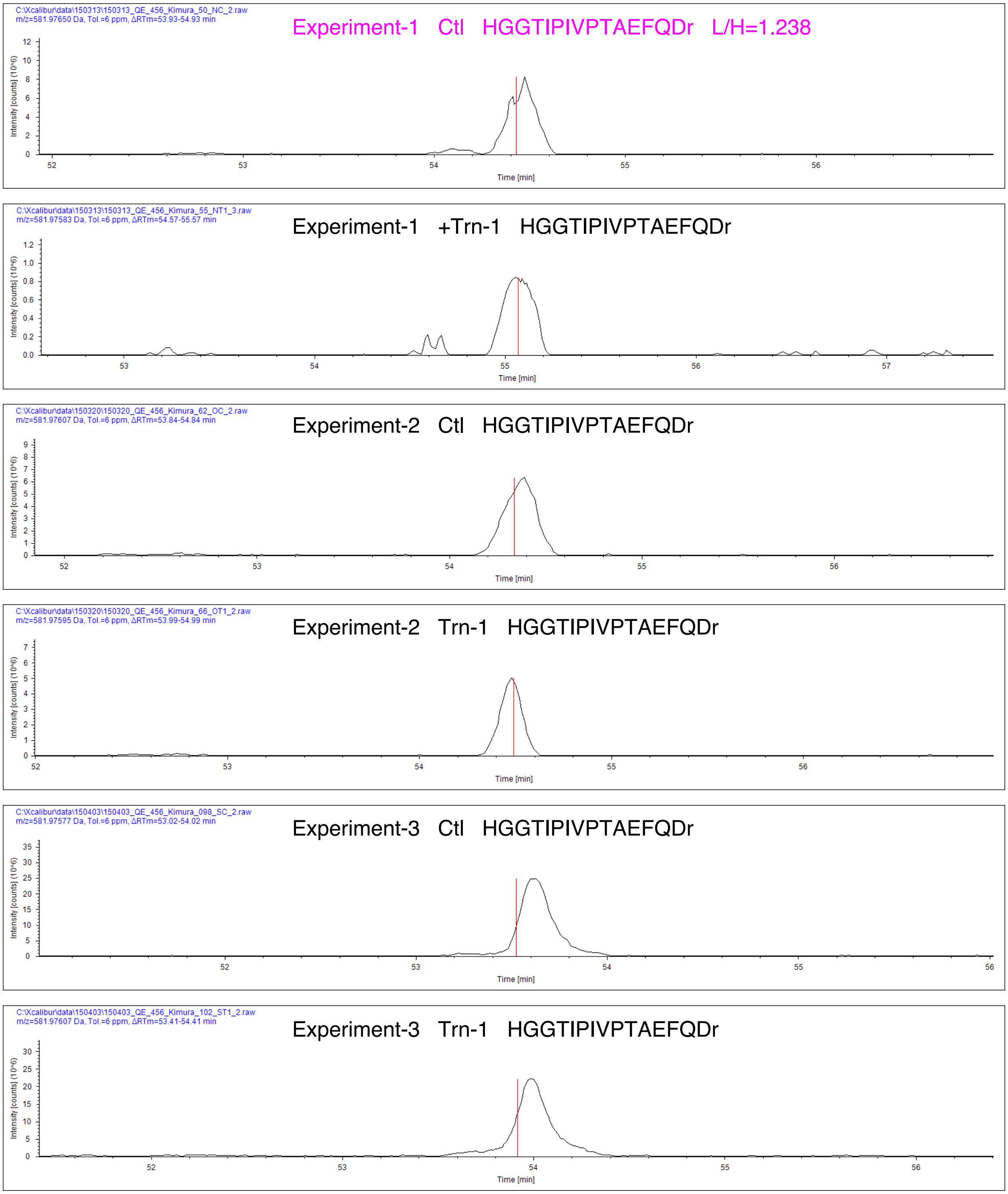

D **ALASLMTYk**

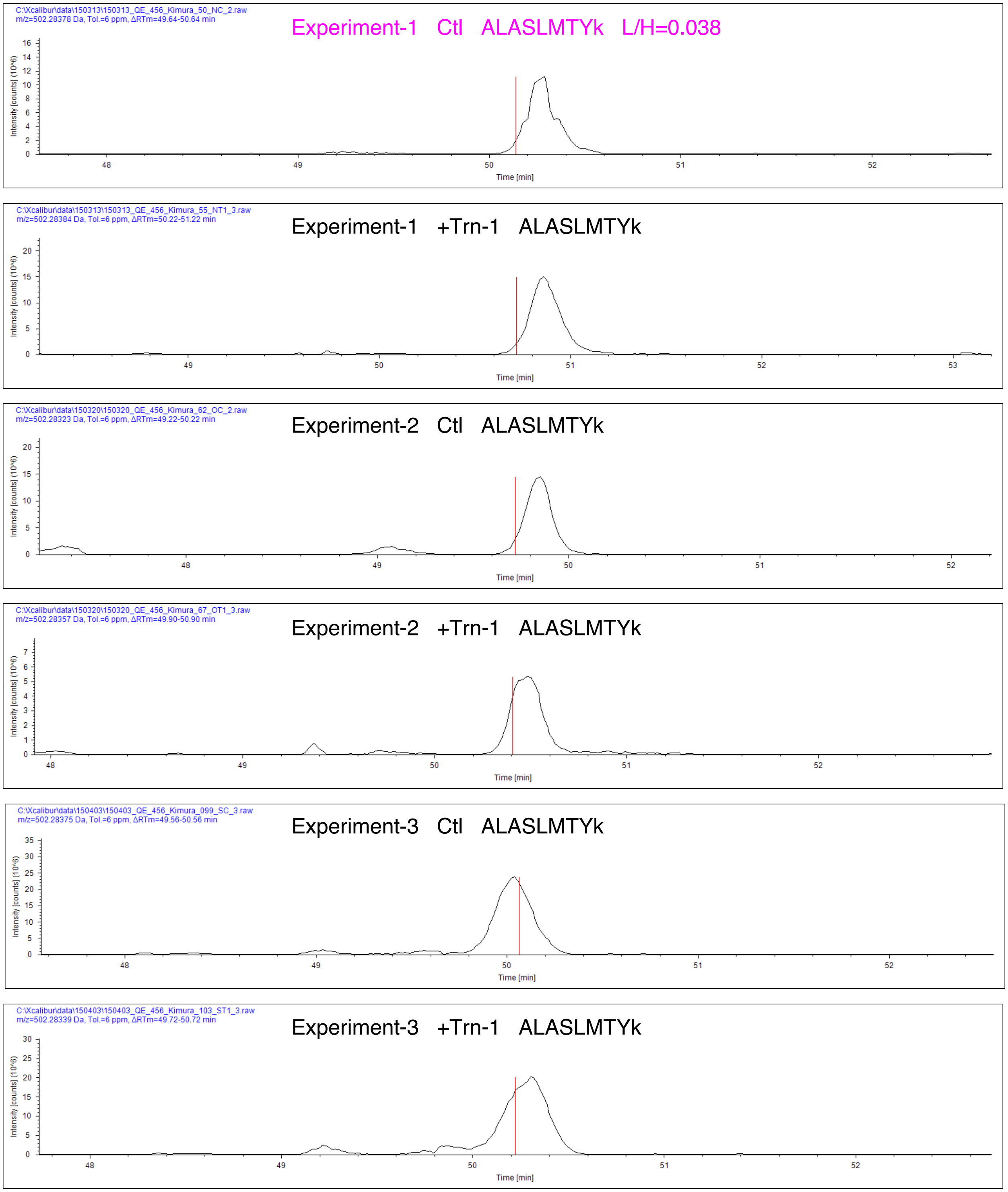
C:\Xcalibur\data\150313\150313_QE_456_Kimura_50_NC_2.raw
m/z=502.28378 Da, Tol.=6 ppm, ΔRTm=49.64-50.64 min
Experiment-1 Ctl ALASLMTYk L/H=0.038
Intensity [counts] (10^6)
Time [min]
C:\Xcalibur\data\150313\150313_QE_456_Kimura_55_NT1_3.raw
m/z=502.28384 Da, Tol.=6 ppm, ΔRTm=50.22-51.22 min
Experiment-1 +Trn-1 ALASLMTYk
C:\Xcalibur\data\150320\150320_QE_456_Kimura_62_OC_2.raw
m/z=502.28323 Da, Tol.=6 ppm, ΔRTm=49.22-50.22 min
Experiment-2 Ctl ALASLMTYk
C:\Xcalibur\data\150320\150320_QE_456_Kimura_67_OT1_3.raw
m/z=502.28357 Da, Tol.=6 ppm, ΔRTm=49.90-50.90 min
Experiment-2 +Trn-1 ALASLMTYk
C:\Xcalibur\data\150403\150403_QE_456_Kimura_099_SC_3.raw
m/z=502.28375 Da, Tol.=6 ppm, ΔRTm=49.56-50.56 min
Experiment-3 Ctl ALASLMTYk
C:\Xcalibur\data\150403\150403_QE_456_Kimura_103_ST1_3.raw
m/z=502.28339 Da, Tol.=6 ppm, ΔRTm=49.72-50.72 min
Experiment-3 +Trn-1 ALASLMTYk

E GASIVEDkLVEDLr

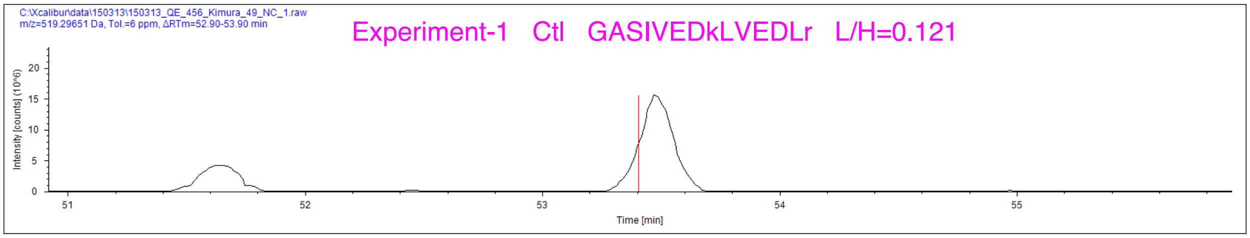

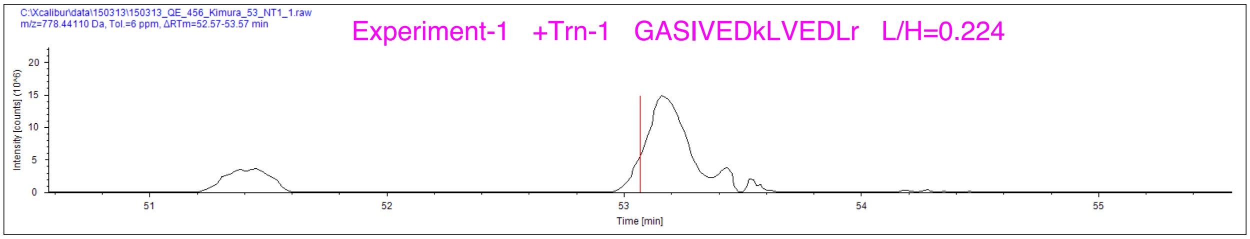

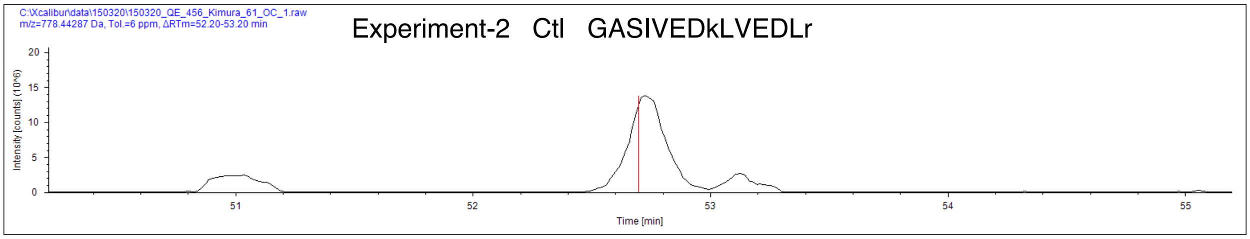

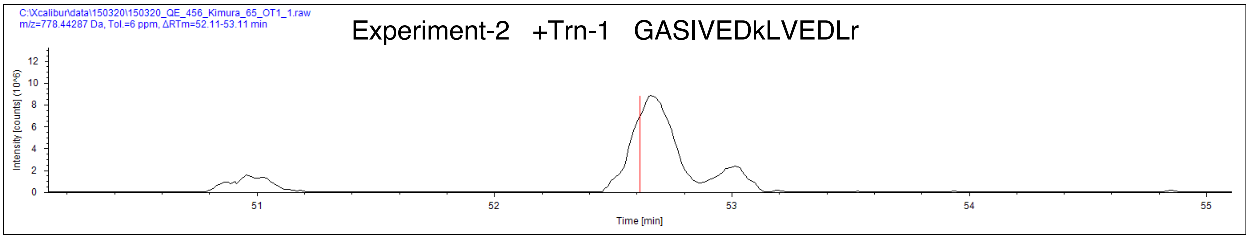

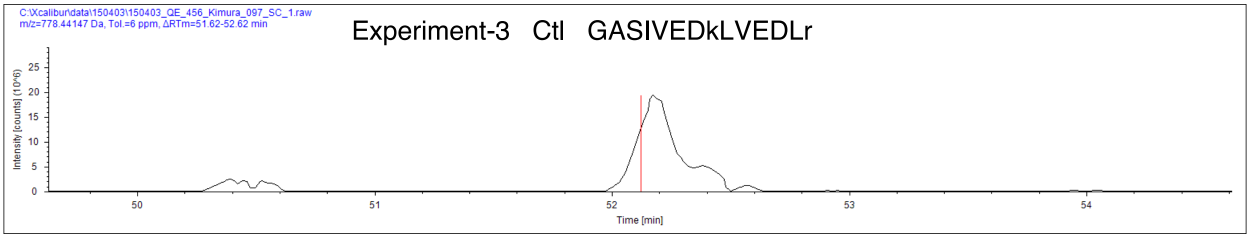

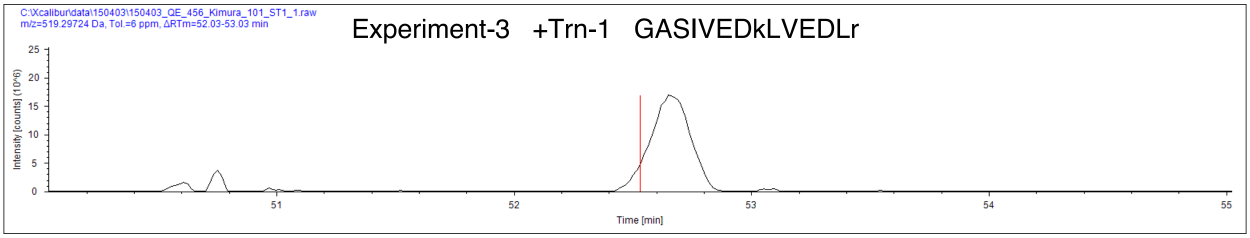

F **TFVVQGFGNVGLHSMr**

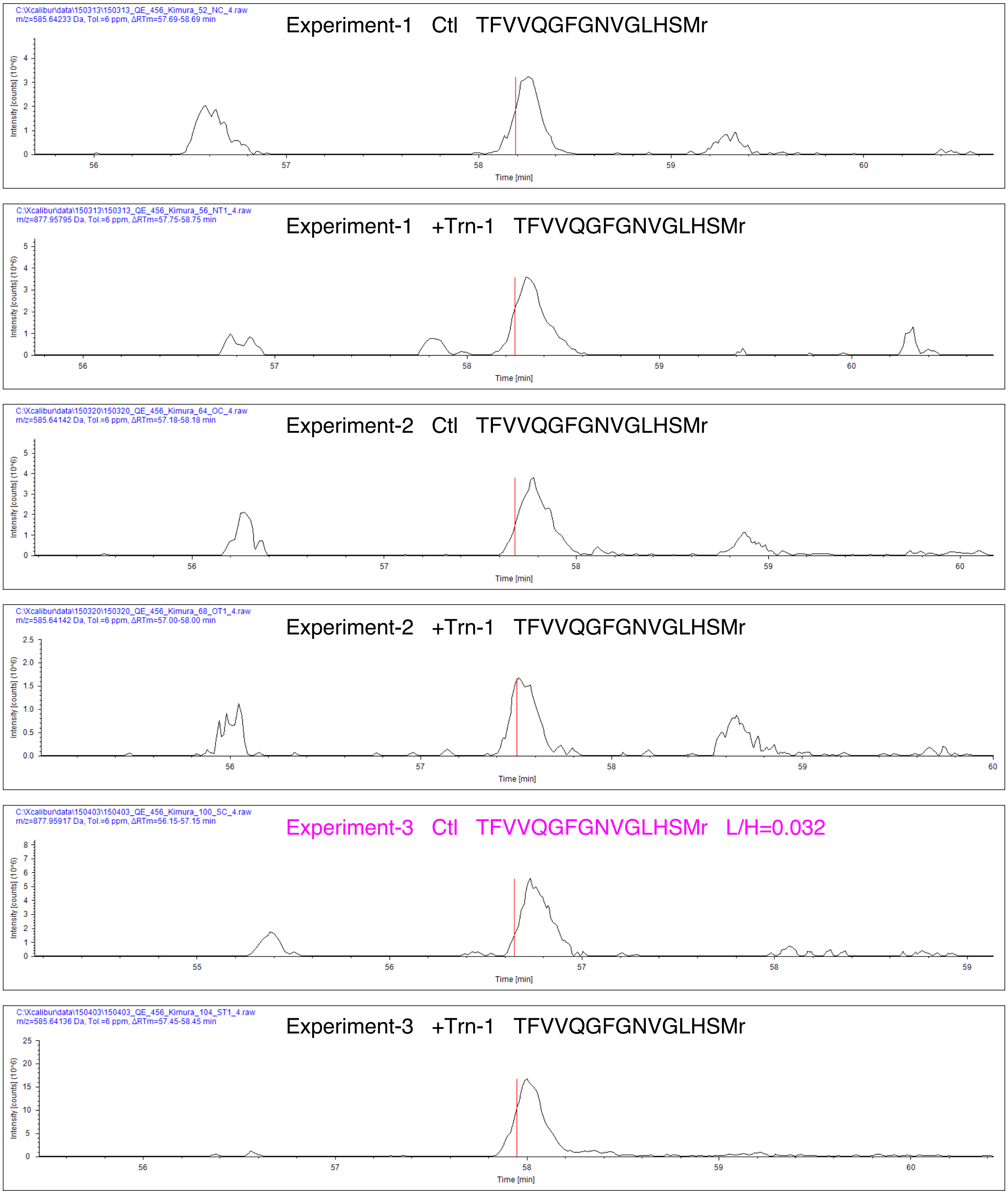

G LTFkYEr

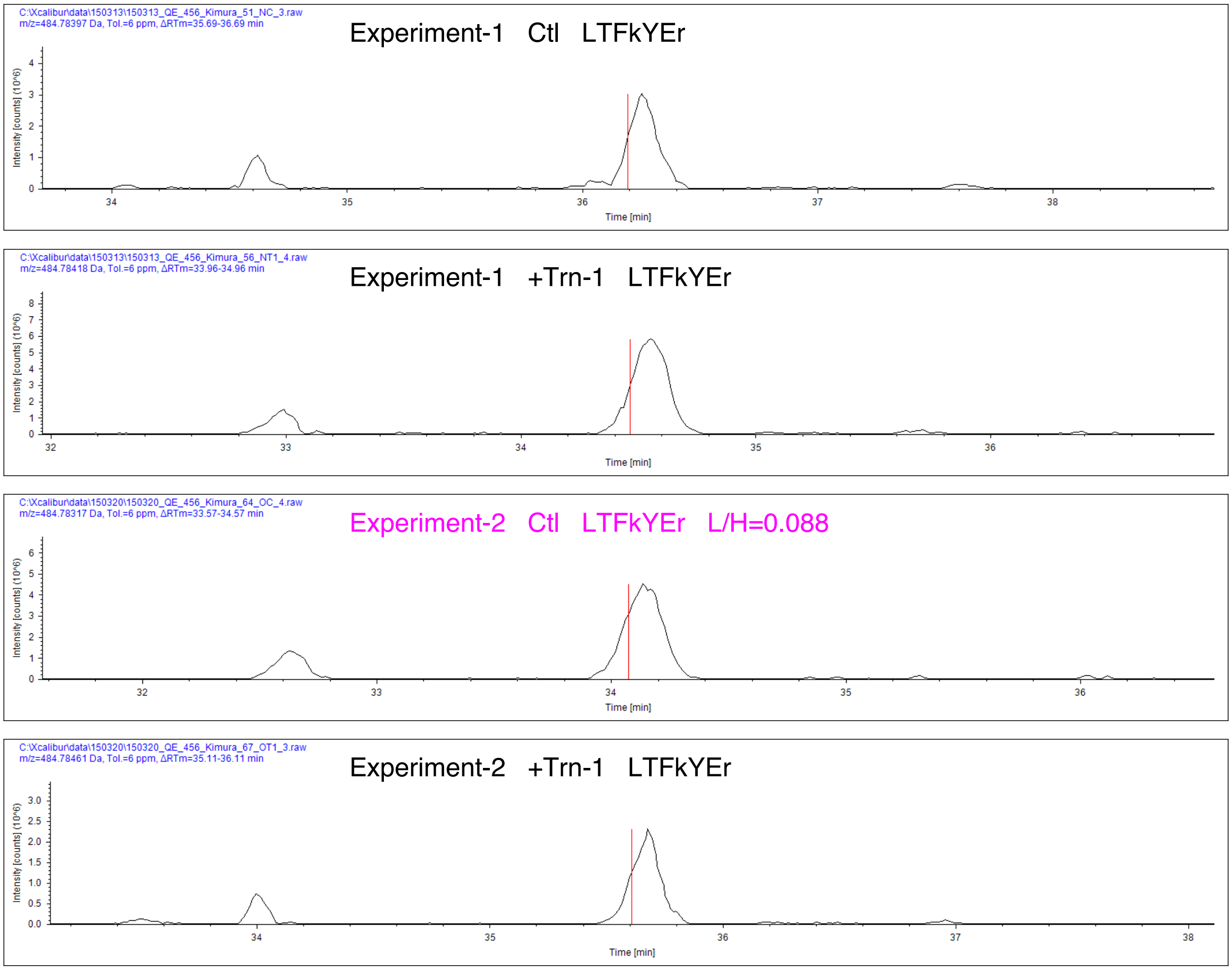

H NYTDNELEk

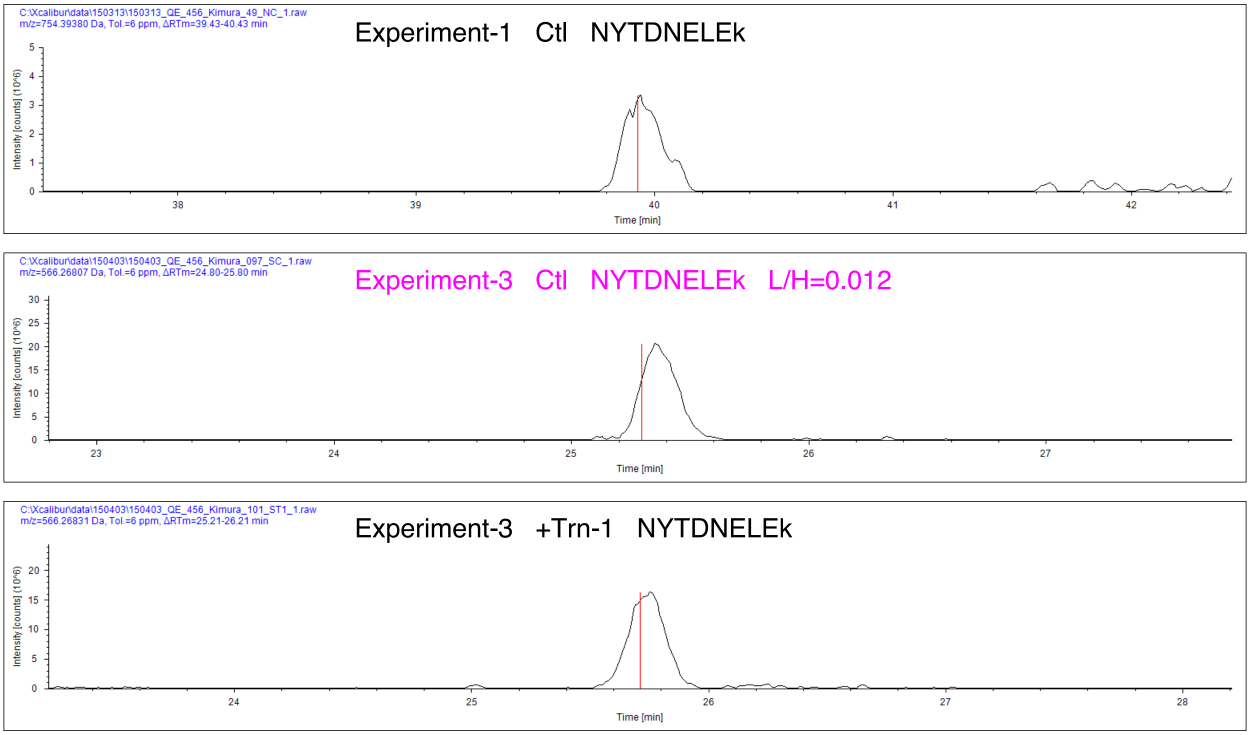

Supplement: Supplementary file 4. — EICs of GLUD1 peptides in the SILAC-Tp with Trn-1: As a general problem of high-throughput LC-MS/MS quantitation, quantitative values of proteins with fewer quantified peptides deviate among the replicates. Referring to EICs of the quantified peptides is useful to avoid misidentification of cargoes. For example, the L/H ratios of a Trn-1 2nd-Z-15% cargo GLUD1 (P00367, ranked 110th and 342nd by the second and third Z-score, respectively) deviated largely in the three replicates of SILAC-Tp with Trn-1 (Supplementary file 1). Panels (A–H) show EICs of the indicated peptide (trypsin targets, K and R, are written in lower cases) in the three (three Ctl and there +Trn) experiments (some peptides were not identified in all the experiments). Magenta letters indicate the quantified peptides and L/H rations. In panel (A), the elution time of the peptide TAMkYNLGLDLr differs largely between the expriment-1 Ctl and experiment-2 +Trn-1, the peak shape of the experiment-2 +Trn-1 is irregular, and the L/H ratio of it is much higher than those of other peptides in +Trn-1 experiments (B and E). Thus, there is concern about misidentification. Because the L/H count of GLUD1 in the experiment-2 +Trn-1 is two (TAMkYNLGLDLr and NLNHVSYGr, A and B) and the L/H ratio of a protein is defined as the median, the L/H ratio of GLUD1 in the expriment-2 +Trn-1 is affected by the L/H ratio of TAMkYNLGLDLr. Exclusion of the L/H ratio of TAMkYNLGLDLr in the experiment-2 +Trn-1 lowers the Z-score rank of GLUD1 significantly. In panel (C), the chromatogram of the peptide HGGTIPIVPTAEFQDr in the experiment-1 Ctl has an irregular peak, and the L/H ratio of it is much higher than those of other peptides in the Ctl experiments (A–H). Thus, overlap with other peptide or other failures may be possible. However, the L/H count of GLUD1 in the experiment-1 Ctl is four (TAMkYNLGLDLr, HGGTIPIVPTAEFQDr, ALASLMTYk, and GASIVEDkLVEDLr) and the value of HGGTIPIVPTAEFQDr does not affect the median. (The L/H ratio of [file elife-21184-supp4.pdf]
